# Fast fabrication of “all-in-one” injectable hydrogels as antibiotic alternatives for enhanced bacterial inhibition and accelerating wound healing

**DOI:** 10.1186/s12951-024-02657-4

**Published:** 2024-07-26

**Authors:** Juan Xin, Zhangyou Yang, Shurong Zhang, Lili Sun, Xin Wang, Yang Tang, Yan Xiao, Honglin Huang, Wei Li

**Affiliations:** 1 Department of Medicinal Chemistry, School of Pharmacy, Chongqing Medical University, Chongqing, 400016 People’s Republic of China; 2 Chongqing Research Center for Pharmaceutical Engineering, Chongqing Medical University, Chongqing, 400016 People’s Republic of China

**Keywords:** Metal–organic framework, Carboxymethyl chitosan, Hydrogels, Nitric oxide, Antibiotic alternatives, Wound healing

## Abstract

Skin wound infection has become a notable medical threat. Herein, the polysaccharide-based injectable hydrogels with multifunctionality were developed by a simple and fast gelation process not only to inactivate bacteria but also to accelerate bacteria-infected wound healing. Sodium nitroprusside (SNP) loaded PCN-224 nanoparticles were introduced into the polymer matrix formed by the dynamic and reversible coordinate bonds between Ag^+^ with carboxyl and amino or hydroxyl groups on carboxymethyl chitosan (CMCS), hydrogen bonds and electrostatic interactions in the polymer to fabricate SNP@PCN@Gel hydrogels. SNP@PCN@Gel displayed interconnected porous structure, excellent self-healing capacity, low cytotoxicity, good blood compatibility, and robust antibacterial activity. SNP@PCN@Gel could produce reactive oxygen species (ROS) and NO along with Fe^2+^, and showed long-term sustained release of Ag^+^, thereby effectively killing bacteria by synergistic photothermal (hyperthermia), photodynamic (ROS), chemodynamic (Fenton reaction), gas (NO) and ion (Ag^+^ and -NH_3_^+^ in CMCS) therapy. Remarkably, the hydrogels significantly promoted granulation tissue formation, reepithelization, collagen deposition and angiogenesis as well as wound contraction in bacteria-infected wound healing. Taken together, the strategy represented a general method to engineer the unprecedented photoactivatable “all-in-one” hydrogels with enhanced antibacterial activity and paved a new way for development of antibiotic alternatives and wound dressing.

**Graphical abstract:**

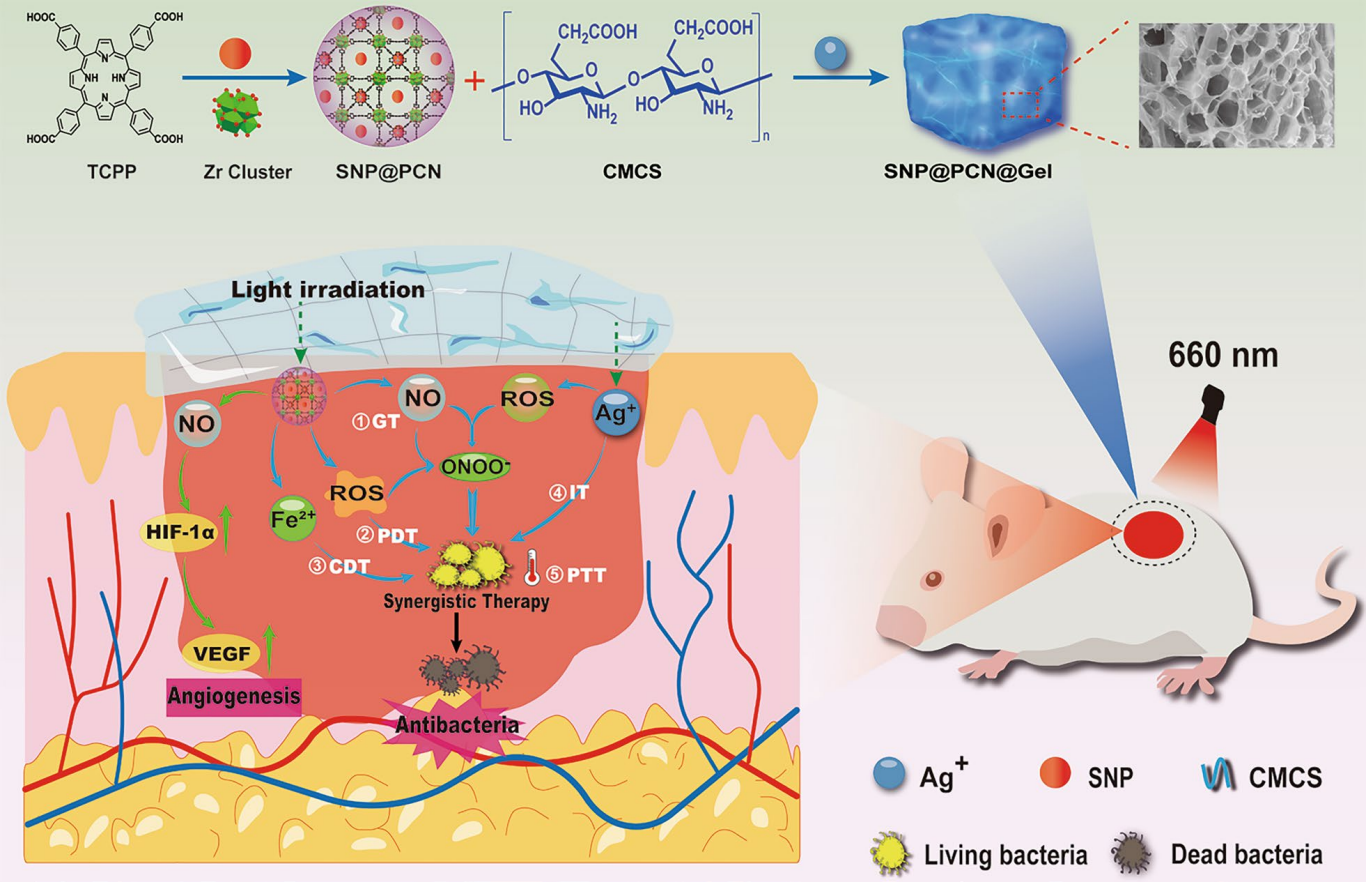

**Supplementary Information:**

The online version contains supplementary material available at 10.1186/s12951-024-02657-4.

## Introduction

Skin prevents the human body from external harm and microbial invasion, and maintains body fluids, nutrients, and electrolytes [1]. However, people often suffered from skin damages caused by various occasional accidents, thus skin wound infection has become a notable medical threat [1]. Currently, antibiotics are still the most widely accepted treatment paradigms for bacterial infections. However, the problem of the expanded use of antibiotics and multidrug resistance is becoming increasingly serious. Therefore, it is urgently needed to develop a new strategy to engineer antibiotic alternatives with both enhanced antibacterial ability and accelerated wound healing without potential safety hazard. So far, various antibiotic alternatives including hydrogels, films, foams, and hydrocolloids have been exploited to satisfy these essential requirements [1–4]. Typically, the burgeoning hydrogels may resolve such unmet needs owing to their unique properties such as good biocompatibility, considerable water retention capabilities, high oxygen permeability, provision of a moist plateau for skin wound healing and a new possibility for fighting bacterial infecting, and acceleration of wound healing [1, 5, 6]. However, it was difficult for traditional hydrogels to access complicated, cramped, and tiny biological interfaces in injured tissue regions due to their fixed shape [5, 7]. Especially, the human body movement might damage their network structure, causing the function loss [5, 6]. If employed in wound dressings, the disrupted hydrogels were easy subject to opportunistic bacteria, resulting in infectious and inflammatory responses in vivo [5, 6]. Recently, syringe-injectable hydrogels have progressed and could be effectively introduced into irregularly shaped sites for wound healing and tissue engineering [5, 7, 8]. Simultaneously, injectable hydrogels with self-healing properties and dynamic reversible bonds (e.g., hydrogen bonds, ion interaction, coordination bond) could prevent the function loss [5]. However, these weaker dynamic reversible bonds often compromised their mechanical performance. Although introducing strong covalent bonds enhanced the mechanical performance [9–11], they caused a trade-off with the injectability, and compromised biocompatibility due to polyacrylic acid or polyacrylamide were non-degradable and cytotoxic [10, 11]. Therefore, hydrogels, as safe and efficient antibiotic alternatives or adjuncts, which have biocompatibility, appropriate mechanical performance, high antibacterial activity and healing efficiency were rare and highly desirable.

Recently, various antibacterial materials including metal/metal oxide materials (e.g., Ag, Au, Pt, ZnO) [12–14], natural biomaterials (e.g., porphyrin, chitosan) [15, 16], artificial nanozymes [17], and antibacterial-nanocarrier system [18, 19], have been put on exploration of novel antibiotic alternatives. Although they have shown promise, some shortcomings still remained. For example, the metal- and organic-based antimicrobial materials often exhibited a short-term efficacy, and created an excessive environment burden due to the rapid active species release [20]. Meanwhile, lack of understanding of the potential toxicity of enzyme mimicry to human beings limited their biomedical applications [17]. Moreover, photocatalytic antimicrobial materials such as zinc oxide, displayed low efficiency due to limited light-absorption ability and low catalytic activity [14, 18, 20]. Therefore, more substantial efforts should be dedicated to exploring highly effective antibacterial materials with excellent biocompatibility, small dosage, and little environment pollution.

Metal–organic frameworks (MOFs) are comprised of metal ions or clusters and organic bridge ligands. Due to well-defined porosity, ultra-high surface area, excellent biocompatibility, and abundant coordination-unsaturated metal sites, MOFs have been widely applied in gas storage, catalysis, and anticancer [21–23]. There also were reports about MOFs employed as antibacterial agent [20]. Especially, PCN-224, a family of Zr-based MOF which is formed by connecting Zr6 clusters and tetrakis (4-carboxyphenyl)-porphyrin (TCPP) ligand, has received increasing attention due to excellent biocompatibility and chemical stability, and adjustable functionality [24]. Notably, porphyrin could efficiently convert 660 nm light to heat for photothermal therapy (PTT) [25], and light to reactive oxygen species (ROS) for photodynamic therapy (PDT) [26, 27] to achieve highly efficient bacterial elimination, because both ROS including ^1^O_2_, hydroxyl radicals(•OH), hydrogen peroxide (H_2_O_2_) and superoxide radicals (O_2_^−^), and hyperthermia could kill bacteria independently by disrupting bacterial cell membranes and DNA [28], or by bacterial protein denaturation and irreversible bacterial destruction [29, 30]. However, ROS had extremely short diffusion length (~ 100 nm in aqueous solution, and < 50 nm within cells and tissues) and lifespan (3.5 μs), thus photosensitizer generally needed to be localized in a close proximity to the focus of infection [30]. Although PTT has many advantages, such as deep tissue penetration, being minimally invasive, and avoidance of drug-resistant bacteria, high power laser and local temperature may be harmful to the surrounding normal tissues [25]. More lethal, the thick extracellular polymers and antioxidant compounds (e.g., glutathione (GSH)) in the mature biofilms blocked the photosensitizer penetration, and deplete ROS, thus severely affecting the efficiency of PDT and PTT [29, 31, 32]. It was found that PDT/PTT (50 °C) combination effectively eradicated biofilms [33], but hyperthermia at 50 °C still had side-effects on the surrounding healthy tissues [34]. Therefore, how to eliminate biofilms by synergistic PDT and low-temperature PTT is still an urgent issue to be resolved.

Nitric oxide (NO)-based on gas therapy (GT) was a potential strategy to combat biofilms formation [34]. NO killed bacteria by lipid peroxidation, DNA cleavage, and protein dysfunction while avoiding antimicrobial resistance [35, 36]. More interestingly, NO could react with ROS to generate ONOO^−^ (peroxynitrite anion) and other reactive nitrogen species (RNS), which exhibited higher biocidal activity than ROS by triggering free radical peroxidation [31, 35–37]. Besides, NO improved the efficiency of PDT and PTT by accelerating intracellular GSH consumption [32], and promoted infected wound healing by improving hypoxia-inducible factor-1α (HIF-1α) protein stability [38] and increasing myofibroblast and collagen generation during skin reconstruction [35]. Other advantages of NO including the far diffusion radius (40–200 μm) and long half-life (ca. 5 s) made it has a large sterilization range which just compensated for the insufficient biocidal area of ROS [26, 34, 36]. Obviously, combining NO-driven PDT with low-temperature PTT may hold a promise for not only reducing the risk of local hyperthermia but also eradicating the mature biofilms. However, endogenous NO may not be sufficient in bacterial infected wounds. Therefore, choosing appropriate NO donor is particularly important for NO-based gas therapy. Sodium nitroprusside dihydrate (SNP) was the most frequently-used NO donor in the clinic. Upon exposure to visible light (390–780 nm), the degradation of SNP might be triggered, spontaneously generating NO and Fe^2+^ [26]. Due to high levels of H_2_O_2_ and more acidic microenvironment in bacterial infected wound [39, 40], the released Fe^2+^ catalytically converted H_2_O_2_ to highly active •OH for effective bacteria-killing, consequently achieving Fenton reaction-based chemodynamic therapy (CDT).

Although using metallic ions as antimicrobial agent face the potential toxicity problems, they still are one of the most utilized antibiotic alternatives. Among them, silver is being extensively used for wound dressings [41–43], due to its excellent antibiotic along with antiviral properties. Especially, using silver does not lead to multidrug resistance and even could accelerate wound healing by cell regeneration in both acute and chronic wounds [41]. It was reported that carboxymethyl chitosan (CMCS) had better solubility, high biocompatibility and water retention, and the plentiful amino groups endowed CMCS with strong and broad-spectrum antibacterial activity [44]. Abundant carboxyl and amino groups can coordinate with metal ions (i.e., Ag^+^, Fe^3+^, Al^3+^), which makes it ideal building blocks of antibacterial hydrogels [44–47]. Ag^+^, as an often-used coordination ion, easily interacts with –OH and –NH_3_^+^ on CMCS to generate hydrogels, conferring CMCS-based hydrogels excellent gelation abilities and antibacterial properties, but with slight unsatisfactory mechanical properties [47]. Notably, the Ag^+^-based hydrogels achieved controllable Ag^+^ release, and maintained durable bacteriostatic effect, thus being conducive to Ag^+^-based ion therapy (IT).

**Scheme 1:**
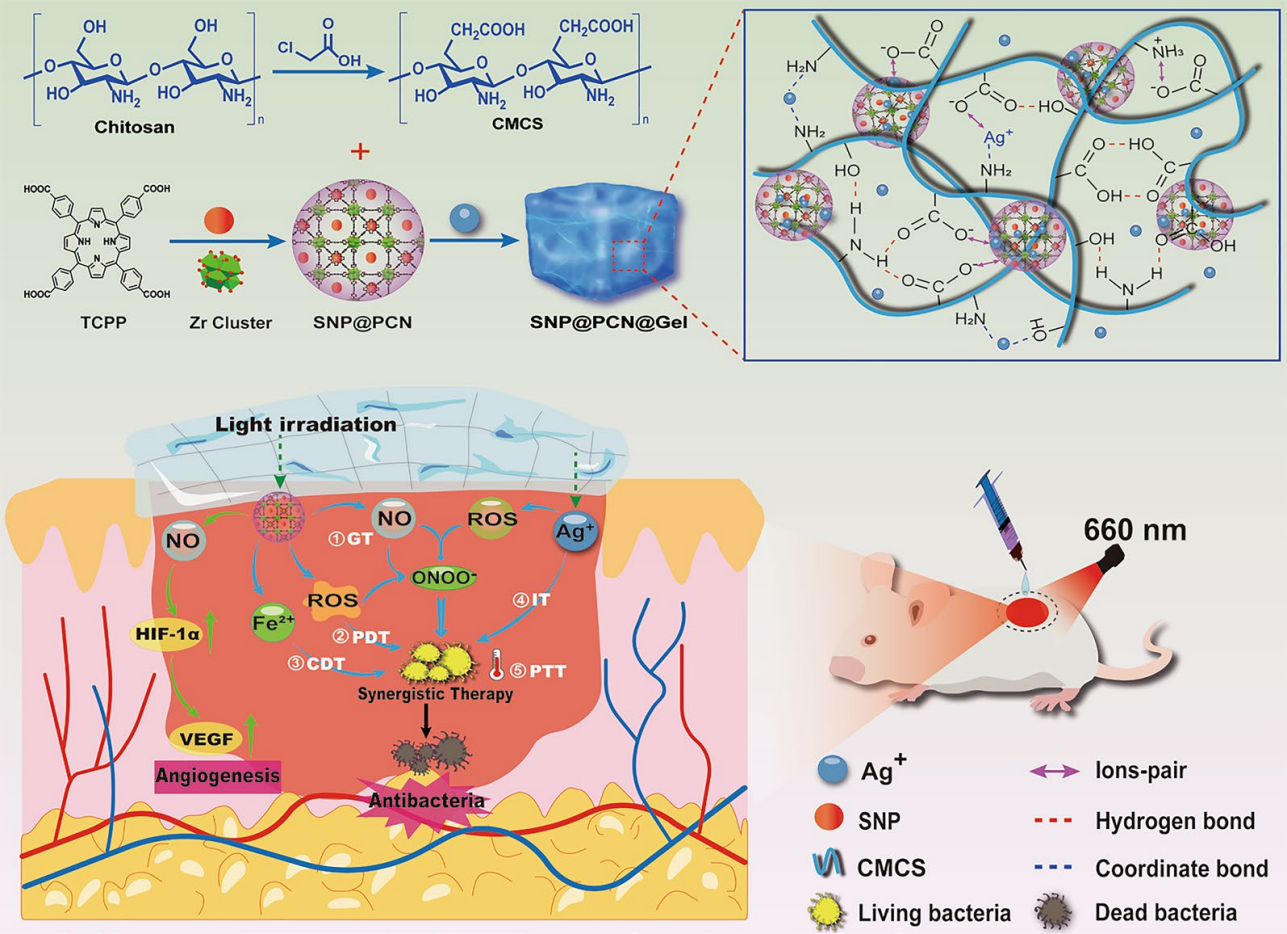
Schematic illustration for the fabrication of SNP@PCN@Gel hydrogels (**A**) and mechanisms of synergistic photothermal therapy (PTT), photodynamic therapy (PDT), chemodynamic therapy (CDT), gas therapy (GT), and ion therapy (IT) (**B**)

Inspired by the fact that SNP can simultaneously release NO and Fe^2+^ under light irradiation, we hypothesized that the combination of Ag^+^-based CMCS hydrogels with SNP-loaded PCN-224 MOF (metal–organic frameworks) nanoparticles would be photoactivatable “all-in-one” therapeutic hydrogels for synergistic photothermal (hyperthermia), photodynamic (ROS), chemodynamic (Fenton reaction), gas (NO) and ion (Ag^+^ and -NH_3_^+^ in CMCS) therapy as illustrated in Scheme 1B. PCN-224 was first synthesized by tetrakis(4-carboxyphenyl)-porphyrin (TCPP) and Zr6 clusters. SNP-loaded PCN-224 nanoparticles were then introduced into the polymer matrix formed by the dynamic and reversible coordinate bonds between Ag^+^ with carboxyl and amino or hydroxyl groups in CMCS, multiple hydrogen bonds and electrostatic interactions in the polymer to fabricate SNP@PCN@Gel hydrogels (Scheme 1A). The hydrogels exhibited shear-thinning and injectable property, and could produce ROS, NO, and ONOO^−^ by cascade reaction under light irradiation. The cascading and synergizing therapy for both fighting Escherichia coli and Staphylococcus aureus infections and accelerating wound healing were validated by in vitro and in vivo experiments. In conclusion, SNP@PCN@Gel could be used as antibiotic alternative and wound dressing.

## Materials and methods

### Materials

All chemicals were used as received. 5,10,15,20-Tetrakis(4-carboxyphenyl) porphyrin (TCPP), zirconyl chloride octahydrate (ZrOCl_2_·8H_2_O), benzoic acid (BA) and terephthalic acid (TA) were from Shanghai Bide Pharmaceutical Co., Ltd. (Shanghai China). N, N-dimethylformamide (DMF), Dimethyl sulfoxide (DMSO) and L-tyrosine (L-tyr) were from Chongqing Chemical Co., Ltd. (Chongqing, China). Penicillin–streptomycin solution (100 ×), fetal bovine serum (FBS), Dulbecco's modified Eagle's medium (DMEM), methyl thiazolyl tetrazolium (MTT), trypsin, and sodium nitroprusside dihydrate (SNP) were from Shanghai Yuanpei Biotechnology Co., Ltd. (Shanghai, China). NO detection kit, ROS assay kit (2,7-dichlorofuorescin diacetate, DCFH-DA), singlet oxygen fluorescent probe (SOSG) and live/dead bacterial double stain kit were purchased from Beyotime Biotechnology (Shanghai, China). Escherichia coli (*E. coli*) and Staphylococcus aureus (*S. aureus*) were obtained from the Agricultural Resources Planning Institute of the Chinese Academy of Agricultural Sciences in the form of agar plate medium. Millipore deionized water (18.2 MΩ cm) was used in all experiments.

### Synthesis of PCN-224

PCN-224 was synthesized according to the reported method [24, 48]. In brief, 10 mL of TCPP solution (2.5 mg/mL in DMF), 5 mL of ZrOCl_2_·8H_2_O solution (15 mg/mL in DMF), and 10 mL BA solution (70 mg/mL in DMF) were added to round-bottom flask and reacted at 90 °C for 5 h under stirring. After that, the obtained mixture was centrifuged at 15, 000 rpm for 10 min to remove unreacted reaction precursor. The collected precipitate was thoroughly washed with DMF three times and dried at 105 °C overnight, PCN-224 was obtained.

### Loading SNP into PCN-224

To load SNP, SNP (40 mg) and PCN-224 (10 mg) were simultaneously resuspended in 4 mL deionized water under stirring at ambient temperature for 24 h [49]. The product was collected by centrifugation (15, 000 rpm, 10 min) and washed with deionized water, obtaining SNP-loaded PCN-224, denoted as SNP@PCN. Drug loading capacity (DLC) was calculated by the following formula:

$$DLC \left(\%\right)= \frac{{m}_{added SNP }- {m}_{unencapsulated SNP}}{{m}_{SNP@PCN}} \times 100 \%$$

where the amount of *m*_unencapsulated_ _SNP_ was equal to that in the supernatant, which was measured by UV–vis spectrophotometer (Shimadazu UV-2600) at 395 nm [38].

### Synthesis of carboxymethyl chitosan (CMCS)

Chitosan suspension (0.1 g/mL in isopropanol, 100 mL) was mixed with 20 mL NaOH solution (10 M) at 60 ℃ and stirred for 1 h. 20 mL of chloroacetic acid solution (0.675 g/mL in isopropanol) was then added and allowed to stir another 3 h at 60 ℃. The reaction mixture was filtrated, washed with ethanol, and dried, obtaining CMCS.

### Synthesis of SNP@PCN@Gel hydrogels

Orthogonal tests which included three factors and five levels were designed to optimize the amount of CMCS, SNP@PCN and Ag^+^ used in the production process of hydrogels. Tabel S1 showed orthogonal test L_25_ (5^3^) parameters, and the detailed values were listed in Table S2. The hydrogels were prepared by adding AgNO_3_ and SNP@PCN to CMCS solution at 70 ℃ under stirring from 10 min. The resulting mixture was cooled, acquiring hydrogels within 10 s, denoted as SNP@PCN@Gel. Blank hydrogels (termed Gel) were prepared as above only without addition of SNP@PCN nanoparticles. The microstructure of hydrogels was analyzed by scanning electron microscopy (SEM, SU8020, Hitachi, Japan).

### Rheological analysis

Rheological analysis was conducted on a modular compact rheometer (MCR 302, Anton Paar, Austria) at room temperature. Dynamic time scanning was carried out for 120 s under 1% stress with a frequency of 10 rad/s. The oscillation stress sweep test was performed at a frequency of 10 rad/s with the stress ranging from 1 to 600%. Shear-thinning tests (10^–2^ to 10^2^ s^−1^, 25 ℃) were performed to determine the viscosity. The recovery scan of SNP@PCN@Gel was conducted at 1% strain for 120 s and 600% strain for 120 s.

### Characterization of PCN-224 and SNP@PCN nanoparticles

The morphologies of PCN-224 and SNP@PCN nanoparticles were observed by scanning electron microscopy (SEM, SU8020, HITACHI, Japan) and transmission electron microscopy (TEM, Thermo Fisher Scientific, USA). The size and polydispersity were also measured using a dynamic light scattering (DLS) system (Zetasizer, Malvern Nano-ZS90). The chemical structure and possible interactions among different components were detected by Fourier transform infrared (FTIR) spectroscopy (Nicolet 6700 iS5, Thermo Fisher Scientific, USA). Spectrum was obtained in the wavenumber ranging from 4000 to 500 cm^−1^ with a resolution of 4 cm^−1^. The crystalline structure was analyzed using X-ray diffractometer (XRD, D8A25, Bruker, Germany) with a detection range from 3 to 30°. N_2_ adsorption and desorption curves at 77 K were determined by Brunauer–Emmett–Teller (BET, Beishide Instrument, 3H-2000PS1, Beijing).

### Photothermal effect test

The photothermal effect was monitored with a digital photothermal imaging system (Testo 875i, Testo Instruments International Trading Co., Ltd, Shanghai, China). Each sample was exposed to 660 nm light (0.4 W/cm^2^) for 15 min, and the temperature was determined at every one minutes. The photothermal stability of SNP@PCN@Gel was further evaluated in four on/off cycles under the same irradiation condition.

### In vitro NO detection

0.1 mL sample was added into 96-well plate and then exposed to 660 nm light (0.4 W/cm^2^) for different period. Then 50 µL Griess Reagent I and 50 µL Griess Reagent II (NO detection kit) were added to 96-well plate and the absorbance was measured at 540 nm [31]. NO concentration was determined using standard curve in supporting materials.

### ROS, ^1^O_2_, ONOO^−^ and •OH detection

To detect the producing efficiency of ROS and ^1^O_2_, 10 µL DCFH-DA probe or 10 µL of 5 μM SOSG probe methanol solution was added into 96-well plate, followed by adding 0.1 mL different sample. Under 660 nm light irradiation (0.4 W/cm^2^) for different period, the fluorescence intensity was determined by a multilabel microplate detection system (PerkinElmer Ltd., MA, USA) at excitation/emission of 488/525 nm for DCFH-DA and 490/520 nm for SOSG [31].

To detect ONOO^−^, 10 mL PBS (0.10 M, pH = 8.2) containing NaHCO_3_ (0.015 M) and L-tyr (5.0 × 10^–4^ M) was prepared at room temperature [27, 31]. Then to the solution was added different samples in the dark or under light irradiation for 10 min. SNP@PCN@Gel-D and SNP@PCN@Gel-L represented the treatment with SNP@PCN@Gel in the dark or under 660 nm light irradiation, respectively. The amount of ONOO^−^ was detected by an F-7000 fluorescence spectrophotometer (HITACHI, Japan).

The •OH-generating capability of SNP@PCN@Gel was determined using nonfluorescent TA as probe due to TA could capture •OH to generate fluorescent compound (2-hydroxyterephthalic acid) with unique fluorescence around 435 nm [50]. To 5 mL of PBS containing TA (0.5 mM) was added 500 mg of samples in the presence or absence of H_2_O_2_ (10 mM), and then incubated for 12 h at 37 °C. Fluorescence analysis was performed by F-7000 fluorescent spectrophotometer (HITACHI, Japan).

### Intracellular ROS and NO detection

According to the previously reported method [51, 52], DCFH-DA and DAF-FM assay kits were used to detect the intracellular ROS and NO, respectively. In brief, *E. coli* (1.0 × 10^6^ CFU/mL) was treated with various samples in the dark or under 660 nm light irradiation for 10 min. After 1 h of incubation, the cells were isolated by low-speed centrifugation, washed with PBS. According to the instruction manual, DCFH-DA and DAF-FM fluorescent probe were added to stain the bacterial for 1 h. The intracellular fluorescence intensity was detected by inversion fluorescence microscope at excitation/emission of 488/525 nm.

### Silver ion release from SNP@PCN@Gel

To investigate Ag^+^ ions releasing behavior of the as-prepared hydrogels, SNP@PCN@Gel (400 mg) was immersed in acetate buffer (pH 5.0) and PBS buffer (pH7.4), respectively, and stirred at 37 °C. 1 mL solution was taken out at 3, 6, 9, 12, 24 and 48 h, equal volume of fresh medium was replaced. The amount of Ag^+^ released was determined by 7700 s inductively coupled plasma mass spectrometer (Agilent ICP-MS, California, USA).

### Cytotoxicity assay

MTT assay was carried out to evaluate the cytotoxicity of hydrogels. Human umbilical vein endothelial cells (HUVECs) were kindly provided by Chinese Academy of Sciences (Shanghai, China) and cultured in DMEM medium with 10% fetal bovine serum and 1% penicillin streptomycin at 37 °C under 5% CO_2_ atmosphere. In brief, the cells were first seeded in 96-well plates at a density of 5000 cells per well and incubated for 24 h. The culture medium was replaced with 200 µL of freshly complete medium containing different concentration of various samples, and then incubated for another 24 h. After remove of culture medium, 20 μL MTT solution (5 mg/mL) was added and incubated for another 4 h. 150 µL of DMSO was then added. the optical density (OD) at 570 nm was determined using a Bio-Rad 680 microplate reader. The cell viability was calculated by the following formula:

$$Cell viability \left(\%\right)= \frac{{OD}_{treated }- {OD}_{blank}}{{OD}_{control }- {OD}_{blank}} \times 100 \%$$

where *OD*_treated_ represented the absence of sample-treated cells, *OD*_control_ represented the obtained OD value in the absence of the sample, and *OD*_blank_ represented the absorbance of culture medium.

### Blood compatibility

To evaluate the blood compatibility of SNP@PCN@Gel, the balb/c mice’s whole blood was withdrawn and employed to prepare a 4% red blood cell suspension. Simultaneously, 9% normal saline and Triton X-100 was used as negative and positive controls, respectively. The experimental group was incubated with SNP@PCN@Gel (0.3125, 0.625, 1.25, 2.5, 5 mg/mL) for 1 h at 37 ℃. After centrifugation (3000 rpm, 10 min), the absorbance was determined at 545 nm by a Bio-Rad 680 microplate reader to evaluate the hemolysis rate.

### In vitro antibacterial activity assay

The antibacterial activity was first assessed by agar-diffusion method [44]. Briefly, the bacteria were overnight incubated in Luria–Bertani (LB) liquid medium. 0.1 mL fresh cultured bacterial solution (1.0 × 10^7^ CFU/mL; CFU = colony-forming units) was applied on the LB solid plates. Various samples (PBS, Gel, PCN@Gel, SNP@Gel, and SNP@PCN@Gel) were placed into a small hole with a diameter of 6 mm in the agar plates without or with exposure to 660 nm light irradiation for 10 min, respectively. The agar plates were incubated at 37 °C for 24 h, and the inhibition zone diameter was measured.

Subsequently, the antibacterial activity was evaluated by plate coating and optical density detection [53]. 0.1 mL of samples (PBS, Gel and SNP@PCN@Gel in the dark or under 660 nm light irradiation for 10 min) was added to 2.0 mL of the bacterial suspensions (1.0 × 10^6^ CFU/mL) and incubated for 2 h at 37 ℃. The optical density was determined at 600 nm by a Bio-Rad 680 microplate reader. The relative viability was calculated using the following formula:

$$Relative viability \left(\%\right)= \frac{{A}_{sample }- {A}_{blank}}{{A}_{PBS }- {A}_{blank}} \times 100 \%$$

where* A*_sample_ was the optical density of bacterial suspensions treated with samples. *A*_blank_ was the optical density of LB medium and* A*_PBS_ represented the optical density of bacterial suspensions treated with PBS.

After these, each group of cultured bacterial suspensions was diluted 1000 times. Part of the diluted suspension (0.1 mL) was spread on LB plates, and then incubated for 24 h at 37 ℃. Finally, the agar plates were photographed and the number of bacterial colonies was recorded.

Further, the morphological changes of bacterial cells were examined using SEM after different treatments for 1 and 3 h at 37 ℃. The bacterial cells were fixed with 2.5% glutaraldehyde overnight, rinsed with PBS and then dehydrated with different concentration of ethanol aqueous solution. SEM images were taken.

### Anti-biofilms analysis

1.0 mL of logarithmic growth period *S. aureus* and *E. coli* solution (1.0 × 10^8^ CFU/mL) was incubated in confocal dish (Beyo Gold 35 mm) for 72 h to form biofilms, and then 0.1 mL of various samples was added to interact with biofilms for 2 h. 1.5 µL SYTO9 and 1.5 µL propidium iodide (PI) dyes were stained for 15 min in the dark. After staining, the living bacteria presented green fluorescence, while dead bacteria showed red fluorescence. Then, the stained biofilms were imaged by a confocal laser scanning microscope (CLSM, Leica TCS SP8, Germany).

### Wound healing assay

A wound healing experiment was adopted by the scratch method to evaluate the migration ability of HUVECs. In brief, HUVECs cells were seeded into 6-well plates at a density of 8.0 × 10^5^ cells per well until the cells reached 90% confluence. A sterilized 10 µL pipette tip was employed to make a straight, parallel and cell free wound in each well. Then, HUVECs cells were treated with PBS, Gel, and SNP@PCN@Gel (1.5 mg/mL) in the dark or under light treatment for 10 min [38], respectively. Cells migration was observed at 0, 12 and 24 h, and the migration rate was calculated by simulating the scratch area ratio of before and after cell migration using ImageJ software [53].

### Tube formation assay

To evaluate the tube formation, 200 μL Matrigel (Corning, USA) was added into 48-well plate and polymerized for 30 min at 37 ℃. HUVECs pretreated with PBS, Gel, and SNP@PCN@Gel (1.5 mg/mL) in the dark or under light treatment for 10 min were inoculated onto Matrigel (5.0 × 10^4^ per well), and incubated. To observer the tube formation, the cells was photographed. The ImageJ software was employed to analyzed the formation and length of the tubes [53]. 

### In vivo antibacterial activity test

All animal experiments complied with the ARRIVE guidelines for Care and Use of Laboratory Animals, and were conducted in accordance with the protocol approved by the Ethics Committee of Chongqing Medical University (Chongqing, China). Balb/c mice (20–25 g, 8 weeks) were provided by the Experimental Animal Center of Chongqing Medical University (SYXK 2022–0016). Mice were randomized into four groups (n = 6 per group [26, 37]). (Control I): PBS; (Control II): blank Gel; (Control III): SNP@PCN@Gel in the dark, donated as SNP@PCN@Gel-D; (Treatment IV): SNP@PCN@Gel plus 660 nm light irradiation, donated as SNP@PCN@Gel-L. To build the infection model, the wounds with 10 mm diameter were made on the back of each mouse under anesthesia, and then infected by 100 μL *S. aureus* suspension (1.0 × 10^7^ CFU/mL) [27]. Sequentially, the wounds were treated with PBS, Gel, and SNP@PCN@Gel in the dark or under 660 nm light irradiation for 10 min. The photographs of the wounds were taken on days 0, 3, 7 and 14 using a digital camera to observe the infection and wound healing process, respectively. To evaluate the anti-infection ability of various samples, the bacteria around the wounds were collected using medical swabs on 14 day and the number of bacteria was measured by the dilution coating plate method [38, 52]. The body weight was recorded every 48 h and the closure rate of mouse wounds was assessed using ImageJ software. During the follow-up treatment process, no mortality occurred.

### Histological analysis

For histological analysis, mice were killed according to the settled schedule. The skin tissue around the wound was carefully harvested, fixed in 4% paraformaldehyde solution, and embedded in paraffin. The tissue specimen was sectioned in the cross-sectional or longitudinal direction and stained with Hematoxylin and eosin (H&E), Masson's trichrome, Sirius red and Immunohisto- chemistry (IHC) staining analysis. Antibodies used in IHC analysis included vascular endothelial growth factor (VEGF), platelet endothelial cell adhesion molecule-1 (CD31), α-smooth muscle actin (α-SMA), hypoxia-inducible factor-1α (HIF-1α), Interleukin-1β (IL-1β), and Interleukin-6 (IL-6). ImageJ software was used for quantitative analysis of each sample. Furthermore, the main organs, including the heart, liver, spleen, lung, and kidney, were carefully collected after 14 days of therapy, and stained with H&E for histological analysis.

### Statistical analysis

All results obtained from experiments were presented as the mean ± standard deviation (SD). Unpaired Student’s t-test was used to analyze statistical significance between two groups. Statistical differences among groups were calculated by one-way analysis of variance (ANOVA). Differences were considered significant when p < 0.05(*), p < 0.01(**), p < 0.001(***).

## Results and discussion

### Preparation and characterization

**Fig. 1:**
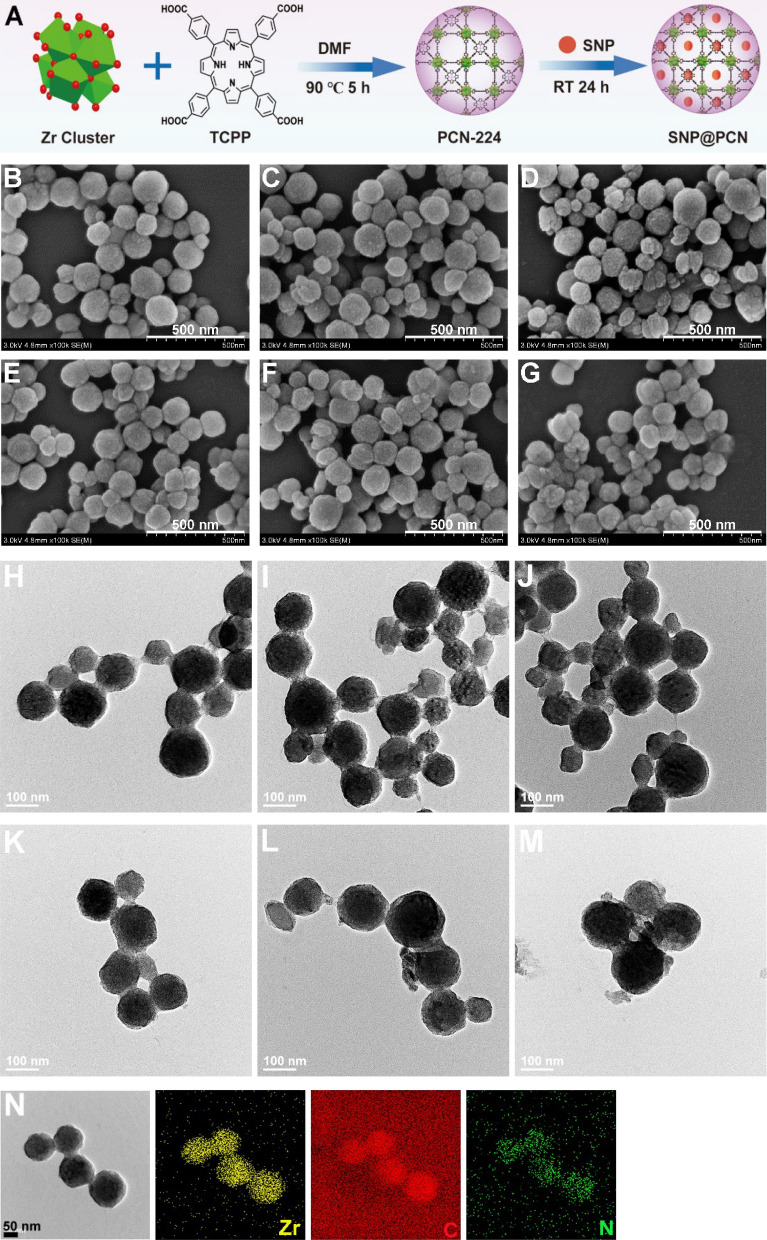

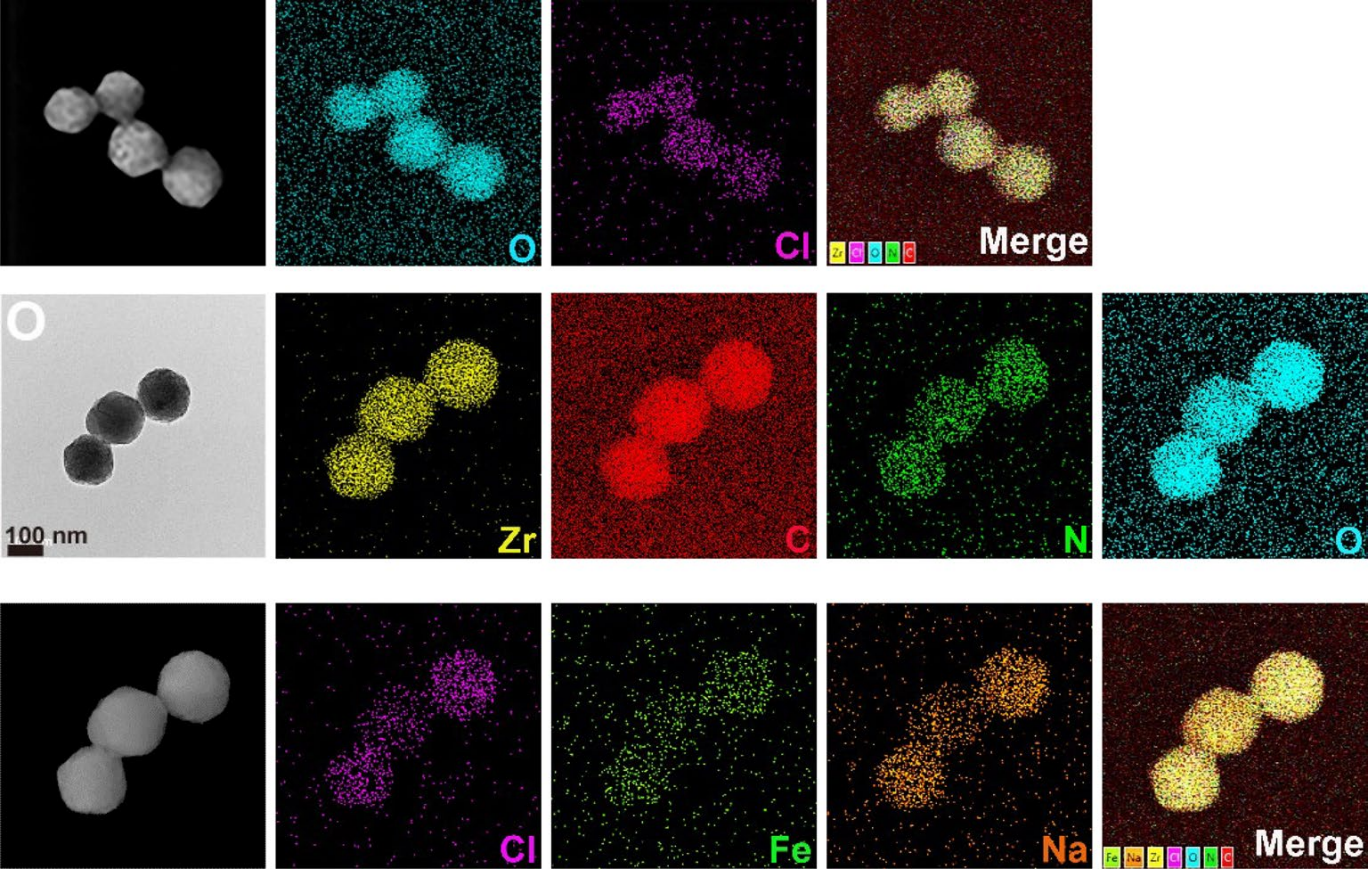
Synthesis and characterization of PCN-224 and SNP@PCN nanoparticles. **A** Schematic diagram showing the synthesis of PCN-224 and SNP@PCN nanoparticles. **B**–**D**, **E**–**G** SEM images of PCN-224 and SNP@PCN nanoparticles after stored at room temperature for 0, 7 and 14 days, respectively. **H**–**J**, **K**–**M** TEM images of PCN-224 and SNP@PCN nanoparticles after stored at room temperature for 0, 7 and 14 days, respectively. **N**, **O** TEM and element mapping images of PCN-224 and SNP@PCN anoparticles, respectively

Based on our design (Fig. 1A), PCN-224 was first synthesized by ZrOCl_2_·8H_2_O, TCPP ligand, and BA in DMF at 90 ℃ according to a previous procedure [48]. The significant advantage of PCN-224 was to employ TCPP as its components rather than encapsulation or adsorption, which prevented TCPP from their aggregation in aqueous solutions [28, 31, 48]. Therefore, TCPP had sufficient contact with surrounding O_2_ molecules, being good for giving high ROS yields. In SEM (Fig. 1B) and TEM images (Fig. 1H), the fabricated PCN-224 exhibited relative sphericity with a size distribution about 100 ± 18 nm, in consistent with the reported results [48], while ~ 90 nm-PCN-224 nanoparticles showed the greatest uptake and best PDT efficacy [48]. After loading SNP, the morphology and size of PCN-224 had hardly changed (Fig. 1E and K, Fig. S1A). The storage stability of PCN-224 and SNP@PCN nanoparticles was also studied by SEM and TEM analysis according to the changes of morphology and size distribution after stored at room temperature for 7 and 14 days. As shown in Fig. 1C–D, I–J and Fig. 1F–G, L–M, the morphology and size did not show significantly change, indicative of good storage stability. However, an increase in the sizes for PCN-224 and SNP@PCN nanoparticles was observed to varied extent in comparation with the characterization results of DLS analysis (Fig. S1B and 1C), probably due to the hydration effect. The phenomena were often observed in other nanoparticles [54, 55]. Elemental mapping (Fig. 1N and O) and energy-dispersive spectrometer (EDS) spectrum (Fig. S2) clearly confirmed all expected elements in the nanoparticles, including Zr, O, N, Cl, Na and Fe, while the presence of Na and Fe elements in SNP@PCN demonstrated that SNP was successfully loaded into PCN-224.

**Fig. 2:**
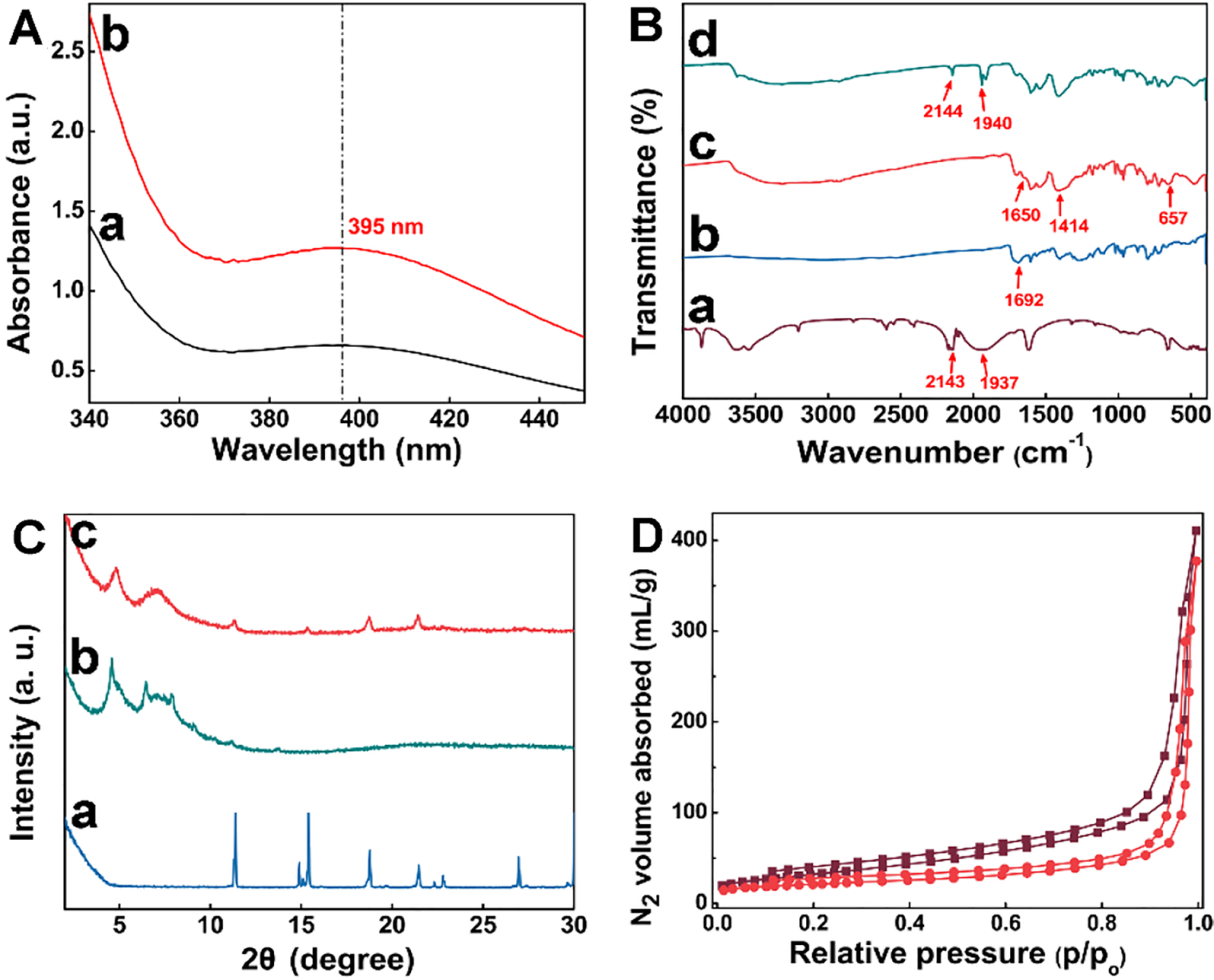
Structure characterization. **A** The Uv–vis absorbance of the supernatant after loading SNP (a) and free SNP (10 mg/mL) (b). **B** FTIR spectrum. (a) SNP, (b) TCPP, (c) PCN-224 and (d) SNP@PCN. **C** X-ray diffractions. (a) SNP, (b) PCN-224 and (c) SNP@PCN. **D** N_2_ sorption isotherms. –■– PCN-224, –●– SNP@PCN

To quantify DLC of SNP@PCN nanoparticles, the amount of SNP in the supernatant after loading SNP was determined by the quantitative standard curve (Fig. S3) and Uv–vis absorption spectra at 395 nm (Fig. 2A), in line with the reported data [38]. 12.7% of DLC was obtained, which was significantly higher than that of either the reported SNP@UCM nanogenerators (4.5%) [38], or SNP@MOF@Au (6.04%) [52]. The high DLC was likely due to the three-dimensional cubic grid-like porous structure of PCN-224 [48], consequently providing sufficient NO donor. FTIR analysis was conducted to confirm the chemical structure (Fig. 2B). Pure SNP showed characteristic FTIR modes (Fig. 2B-a) at 3500–3700 cm^−1^ due to δ-OH of lattice water (symmetric & antisymmetric), 2143 cm^−1^ attributed to υ-CN (axial and equatorial CN ligands), and 1937 cm^−1^ ascribed to the tensile vibration of -NO, a typical band related to the SNP [52]. For TCPP (Fig. 2B-b), the characteristic peak at 1692 cm^−1^ was assigned to the asymmetric vibration band of carboxyl group [56], while PCN-224 exhibited the symmetric and asymmetric stretching vibrational peaks of COO^−^ at 1650 and 1414 cm^−1^ [31, 56], mainly owing to that -COOH in TCPP became -COO^−^ in PCN-224, and the characteristic peak at 657 cm^−1^ was attributed to the stretching vibration of Zr-OH [56], suggesting that PCN-224 was successfully formed by self-assembly of Zr6 clusters and carboxyl in TCPP (Fig. 2B-c). Meanwhile, these typical characteristic peaks (at 2143 and 1937 cm^−1^) of SNP appeared in the FTIR spectrum of SNP@PCN (Fig. 2B-d), demonstrating that SNP had been successfully encapsuled into PCN-224. This result was further confirmed by XRD. As shown in Fig. 2C-b, the characteristic diffraction peaks of PCN-224 were in good consistent with the reference data [25, 48]. The present diffraction peaks at 2θ = 11.0°, 14.8°, 15.5°, 18.7°, 21.4°, 22.8° and 26.9° suggested a high crystallinity of SNP (Fig. 2C-a). Compared with SNP alone, these characteristic peaks of SNP disappeared in the diffraction pattern of SNP@PCN (Fig. 2C-c), implying that SNP was transformed into an amorphous state. Despite a slight decrease in the crystallinity after the loading of SNP was observed, the peaks at 2θ = 4.7° and 6.8° which represented the (002) and (022) crystal planes of PCN-224 [25, 48], respectively, were still found (Fig. 2C-c), indicating the frameworks of PCN-224 were still retained. The porosity of PCN-224 and SNP@PCN nanoparticles was evaluated by nitrogen adsorption and desorption studies at 77 K (Fig. 2D). A nitrogen uptake of 410 mL/g (STP) and Brunauer- Emmett-Teller (BET) surface area of 117.691 m^2^/g have been obtained for PCN-224, larger than that of the reported data (72.4852 m^2^/g and 61.393 m^2^/g [25]), respectively. The nitrogen adsorption exhibited a typical type-I isotherm, in agreement with the crystal structure. The total pore volume of PCN-224 was as high as 0.5903 mL/g, maybe ascribing to the three-dimensional large open channels in the framework [48, 57] consequently making most of the pore surface accessible. For SNP@PCN nanoparticles, it’s STP, BET surface area, and total pore volume was 377 mL/g, 72.4852 m^2^/g, and 0.5211 mL/g, respectively. The element mapping (Fig. 1N and O) in combination with the above-mentioned characterizations suggested the successful synthesis of the SNP@PCN nanoparticles.

**Fig. 3:**
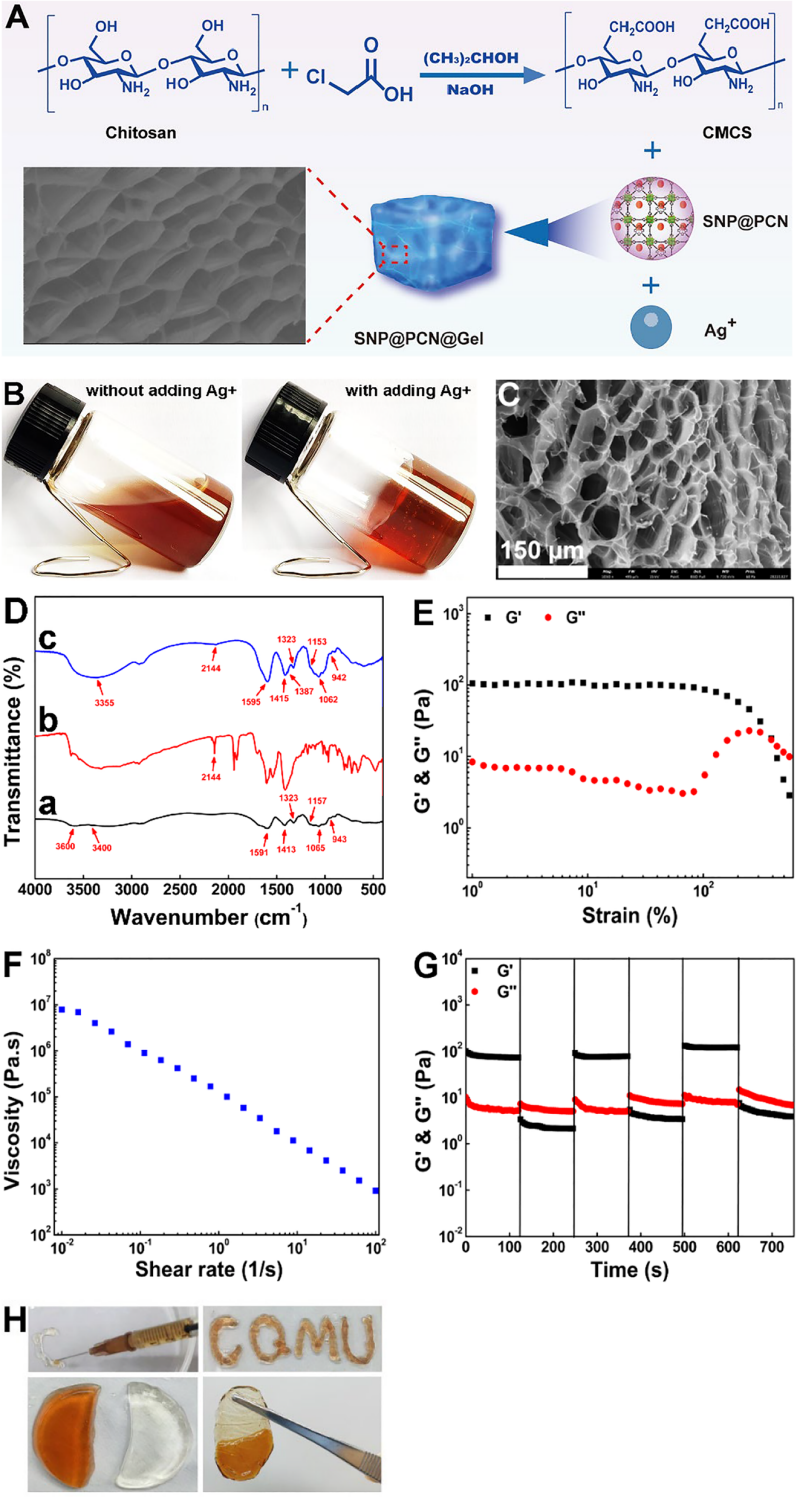
Preparation and characterization of SNP@PCN@Gel. **A** Schematic diagram of SNP@PCN@Gel preparation. **B** Optical images confirming the progress of gelation. **C** SEM images of SNP@PCN@Gel. **D** FTIR of CMCS (a), SNP@PCN (b), and SNP@PCN@Gel (c). **E** Strain sweep measurement of G’ (storage modulus) and G’’ (loss modulus). **F** Shear-thinning test of the SNP@PCN@Gel varying from 0.01 to 100 1/s at 25 °C. **G** Alternate strain sweep of SNP@PCN@Gel. **H** Photograph of injectable hydrogels and letters “CQMU” written by extruding SNP@PCN@Gel from a needle, and the self-healing performance

CMCS has a greater sorption capability for metal ions (*i*.*e*., Ag^+^, Zn^2+^), mainly due to the abundant chelating groups (*i*.*e*., amino, hydroxyl and carboxyl groups) and increased chain flexibility [45]. Notably, compared with the traditional dressings, CMCS exhibited an enhanced capability to heal chronic wounds and burns, mainly due to its hydrophilicity and wettability and provision of an ideal environment for the cell adhesion and pain relief [45, 58], consequently making CMCS excellent candidate to prepare antibacterial hydrogels [44–47]. However, the toxicity of cross-linking agents and complicated preparation procedure restricted the application of CMCS-based hydrogels [59]. Ag^+^ displayed good coordination capability, being suitable for the generation of coordination hydrogels. Therefore, a straightforward and fast gelation procedure was developed just by mixing CMCS, Ag^+^ and SNP@PCN nanoparticles via cross-linking of coordination bonds between Ag^+^ and carboxyl groups (Fig. 3A). The orthogonal experiment was performed to optimize the dosage of each component, and the detailed parameters were listed in Table S2. As shown in Fig. 3B and Fig. S4, we found that only test No. 8, 9, 10, 14, 15 and 19 was able to form hydrogels. The inner geometry was observed via SEM images (Fig. 3C and Fig. S5). It was found that test No. 14 exhibited better inter-connected porous structure (Fig. 3C). Based on these results, the optimal dosage was 25 mg/mL, 0.4 M, and 0.5 mg/mL for CMCS, Ag^+^, and SNP@PCN, respectively. Noting once the mixing was stopped, soft and elastic hydrogels (donated as SNP@PCN@Gel) were formed (within 10 s) (Fig. 3B). Compared with the conventional chemical cross-linking hydrogels [60, 61], the fast gelation process might be ascribed to the strong multiple associations including coordination action, hydrogen bonding and electrostatic actions in the hydrogels. Firstly, thanks to the porous structure and unreacted carboxylic groups, SNP@PCN provided large available surface area (Fig. 2D) and multiple binding sites for Ag^+^ [62]. When Ag^+^ was mixed with SNP@PCN, Ag^+^ infused into SNP@PCN due to electrostatic attraction between Ag^+^ and the carboxyl groups in TCPP. Next, the Ag^+^-infused SNP@PCN adsorbed CMCS by the electrostatic and coordination interactions between the carboxyl groups and Zr6 clusters due to CMCS was negatively charged and had abundant carboxyl groups. Subsequently, the CMCS-coated SNP@PCN were further cross-linked with the surrounding CMCS by the interwoven hydrogen bonding, electrostatic actions between carboxyl groups and Ag^+^, and coordination actions between Ag^+^ and hydroxy or amino groups [62, 63]. These interactions collectively acted to promote SNP@PCN@Gel formation. The added SNP@PCN acted as cross-linking points and physical reinforcements, which also enhanced the mechanical property of CMCS-based hydrogels [63]. The formed inter-connected porous structure was of significance to wound healing, because the porous structure was good for cell adhesion, nutrient supply, gas exchange, and blood and tissue exudates to be rapidly absorbed into the hydrogels [2, 45, 64].

In Fig. 3D-a, CMCS exhibited four characteristic regions at 1064 cm^−1^, 1410 cm^−1^ and 1594 cm^−1^, 1324 cm^−1^ and 2921 cm^−1^, and 3376 cm^−1^[45, 65]. The peaks at about 1160 cm^−1^ and 945 cm^−1^ were ascribed to the typical stretching vibration of C–O–C and the antisymmetric out-of-plane ring stretch of sugar [62]. The band of C≡N stretching vibrations at about 2144 cm^−1^ arose and was well-agreeing with the reported results [52] (Fig. 3D-b). These distinguished bands were also observed in FTIR of SNP@PCN@Gel (Fig. 3D-c), suggesting that the characteristic groups in CMCS did not change after the complexation with Ag^+^. However, compared with pure CMCS, the characteristic signals at 1410 cm^−1^ and 1593 cm^−1^ ascribed to the symmetric and asymmetric stretching vibrations of COO^−^ [45] shifted to 1415 and 1596 cm^−1^, and became more obvious likely due to the interaction between carboxyl groups and Ag^+^ [46]. Meanwhile, the peak at about 3600 cm^−1^ correspond to the O–H and N–H stretching vibration also shifted to 3355 cm^−1^ and a new distinguished signal at about 1387 cm^−1^ also appeared. These observations implied that the probable interactions such as hydrogen bonding and the electrostatic interactions existed in SNP@PCN@Gel, because when hydrogen bonding was formed, the stretching vibration peak of O–H often exhibit typical red-shift [66].

The rheological properties were evaluated by quantitative (Fig. 3E–G and Fig. S6-8) and direct visual methods (Fig. 3H). The critical strain was first assessed by determining the storages modulus (G′) and loss modulus (G″) using a rheometer through the dynamic strain sweep test. In Fig. S6, the G′ was obviously larger than G″, demonstrating that all the hydrogels were in gel state and had an elastic network. Especially, as shown in Fig. 3E for No. 14 hydrogels, the intersection point was at a strain of 388%, and the gel-sol transition occurred at stress values over the yield stress, mainly due to the collapse of its network structure (G′ < G″) [46]. The initial viscosity of SNP@PCN@Gel was 7.83 × 10^6^, higher than that of other hydrogels (Fig. S7), as expected, then dramatically decreased with the increase in the shear rate (Fig. 3F), indicative of a shear-thinning behavior due to the disruption of reversible interactions in the hydrogels network. The self-healing capacity of SNP@PCN@Gel was further evaluated by a cyclic dynamic alternative strain sweep experiment. As shown in Fig. 3G and Fig. S8, all hydrogels maintained intact network at the low shear strain. However, the hydrogels were disrupted by the high strain with an inversion of G″ exceeding G′ (Fig. 3G). When subjects to 600% strain, the value of G′ decreased to about 3.41 Pa, lower than that of G″ (7.32 Pa). SNP@PCN@Gel lost its viscoelastic properties due to the reversible interactions were destroyed, therefore converting into the solution state. However, SNP@PCN@Gel rapidly self-healed and fully recovered back to its original viscoelastic properties (the network of hydrogels) after lowering the strain from 600 to 1%, indicative of excellent self-healing capability [46, 67]. Importantly, this reconstruction behavior was fully reproducible as evidenced by the cyclic tests (Fig. 3G). Considering that the shear-thinning and self-healing capability has been demonstrated, the foundation has been laid for injectability of SNP@PCN@Gel. To visually demonstrate the self-healing and injectability, SNP@PCN@Gel were injected onto a dish by syringe, and used to write the letters “CQMU” (Fig. 3H), presenting a good writing ability. When a piece of SNP@PCN@Gel and bank hydrogels was put together, they finally merged into a whole piece in a short period later, and the fused hydrogels could be picked up (Fig. 3H), confirming its self-healing capability and injectability. Noting dynamic cross-linked hydrogels might exhibit liquid like properties to access complicated, cramped, and tiny biological interfaces in injured regions under natural conditions due to their movable inner structure [46]. The significant convergence of properties demonstrated that SNP@PCN@Gel was suitable for developing hydrogels-based self-adapting wound dressings.

### Evaluation of photothermal conversion and ROS/NO release

**Fig. 4:**
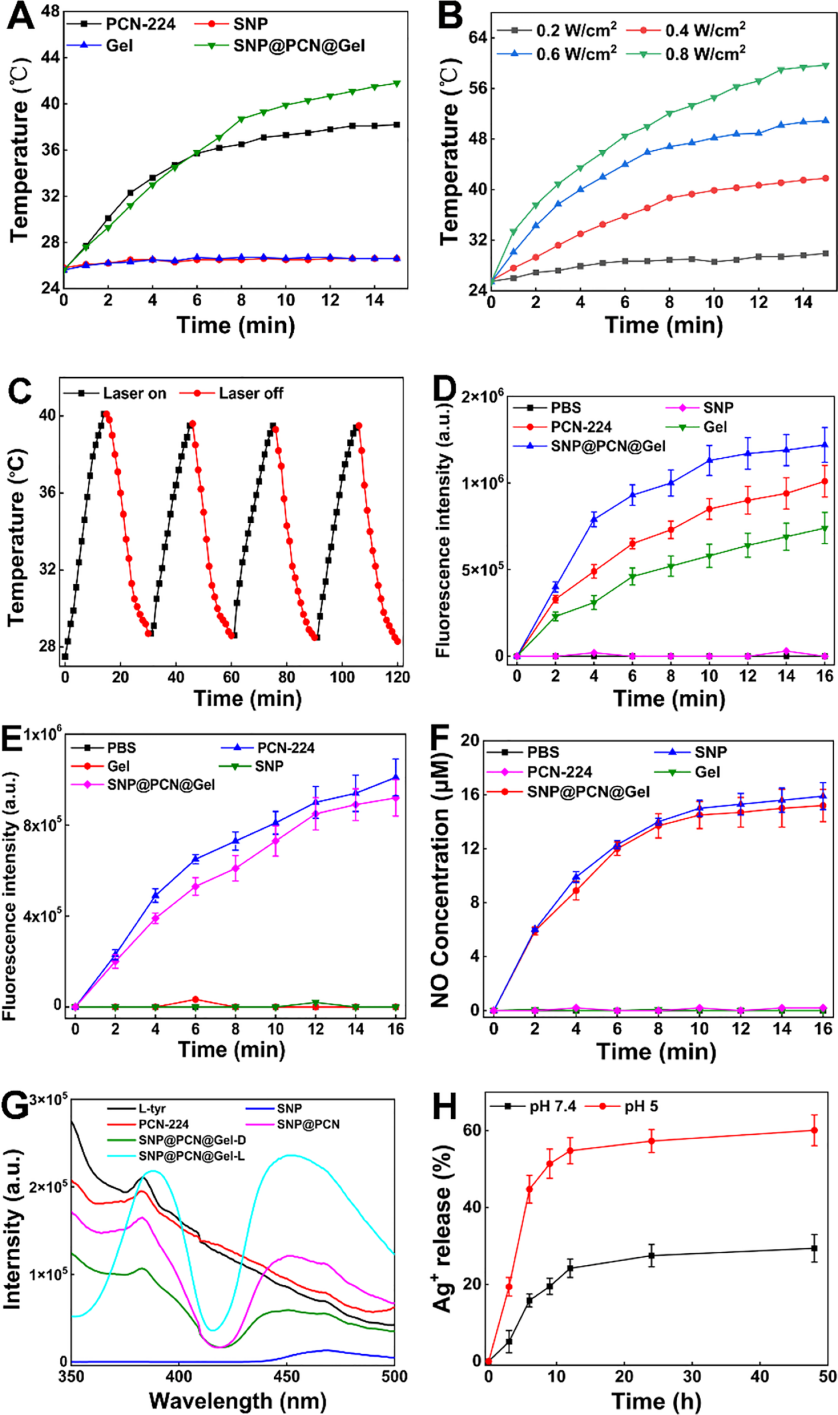

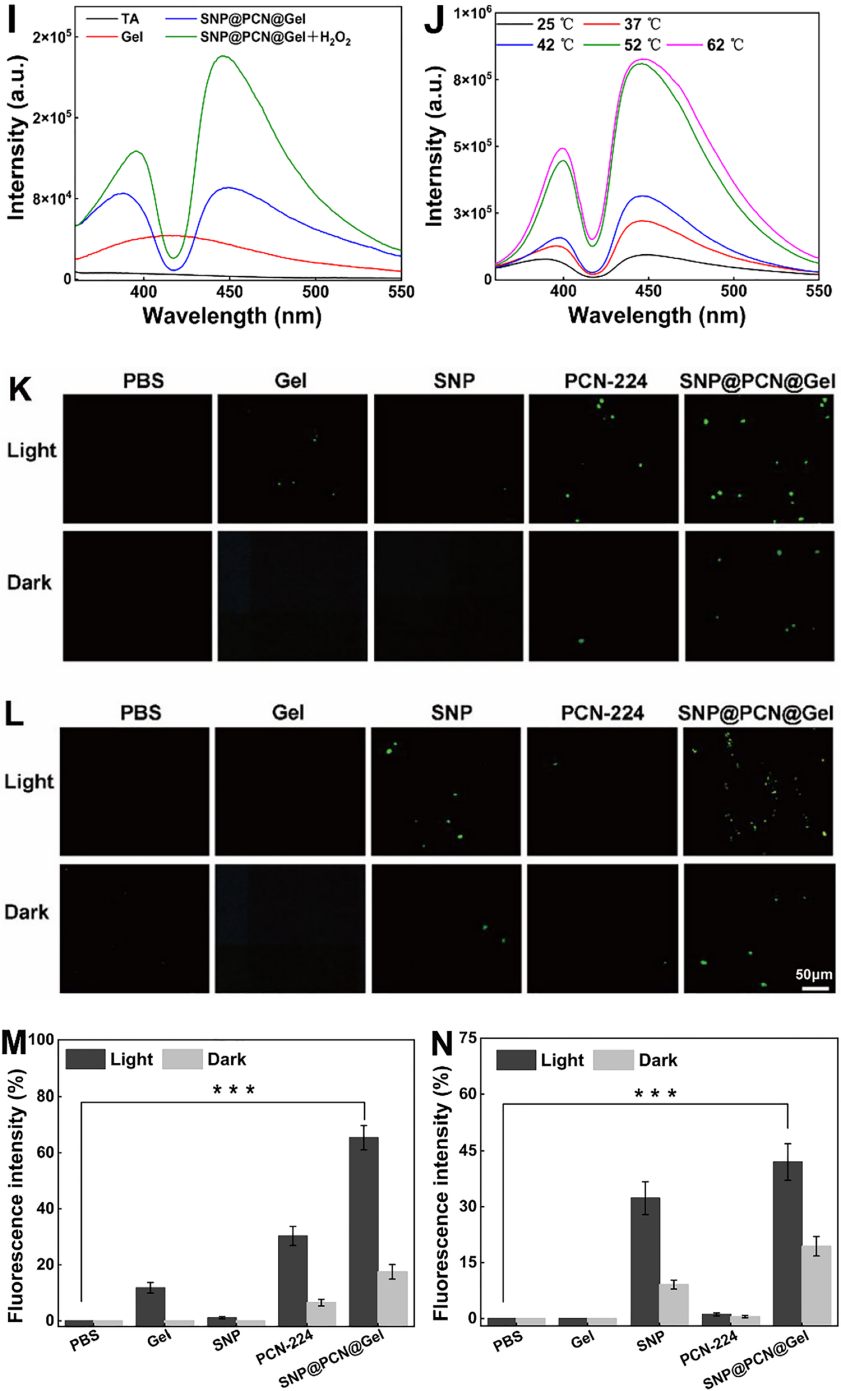
**A** The temperature change of various samples under laser irradiation (0.4 W/cm^2^). **B** The power-dependent temperature change of SNP@PCN@Gel with different power laser irradiation. **C** “On–off” temperature change of SNP@PCN@Gel under laser irradiation (0.4 W/cm^2^). **D** ROS produced by various samples under laser irradiation at different times. **E** ^1^O_2_ produced by various samples under laser irradiation at different times. **F** NO produced by different samples under laser irradiation at different times. **G** ONOO^−^ produced by different samples under laser irradiation. **H** Release of Ag^+^ from SNP@PCN@Gel at pH 5.0 (acetate) and 7.4 (PBS). **I** OH produced by SNP@PCN@Gel using TA as a fluorescent probe. **J** The effect of temperature on the production of OH. **K** and **L** Fluorescence images of intracellular generation ROS and NO in the dark and under light irradiation, respectively. **M** and **N** Fluorescence intensity of intracellular generation ROS and NO in the dark and under light irradiation, respectively. ***p < 0.001

To confirm if SNP@PCN@Gel was suitable for PTT, the photothermal property was evaluated comprehensively. A time-dependent temperature change was observed (Fig. 4A). The surface temperature rapidly increased from 25.0 to 41.8 ℃ for SNP@PCN@Gel within 15 min under 660 nm light irradiation (0.4 W/cm^2^), whereas it only slightly increased to 26.5 °C and 26.7 °C for SNP and blank Gel, respectively. Specifically, PCN-224 and SNP@PCN@Gel showed a similar heating trend after laser irradiation, suggesting that the photothermal conversion capability of PCN-224 was not weakened by SNP loading and gelatin covering. Additionally, the change of temperature displayed a power-density-dependent photothermal performance (Fig. 4B). After four cycles of heating–cooling, SNP@PCN@Gel still kept outstanding photothermal ability under laser irradiation (Fig. 4C). It was well known that PCN-224 had the excellent ROS generation performance under light irradiation [26, 68]. DCFH-DA and SOSG fluorescent probe were used to detect the total ROS and ^1^O_2_ generation efficiency, respectively. As shown in Fig. 4D and E, the fluorescence intensity of PCN-224 and SNP@PCN@Gel at the same amount of TCPP was analogous, confirming that both total ROS and ^1^O_2_ generation efficiency of TCPP were not attenuated by Zr_6_ clusters incorporation and gelatin covering, in line with the reported data [31]. And their generation performance showed time-dependent characteristics. In comparation with ROS which nonspecifically triggered apoptosis and necrosis, NO could prevent oxidative damage of host cells, generate ONOO^−^ for higher biocidal activity and even against antibiotic-resistant bacteria [31, 32, 37]. SNP has been approved as a clinical therapeutic drug and was the most commonly available NO donor. To evaluate NO and subsequently ONOO^−^ generation capability, Therefore, NO and ONOO^−^ were detected with use of Griess reagent and indicator (L-tyr), respectively [31, 34]. With the assistance of the standard curve in Fig. S9A, NO generation was detected (Fig. 4F). The NO generation showed light-triggered characteristics with time-dependence, and there was only little NO production without light irradiation, suggesting the importance of light irradiation (Fig. S9B). As expected, SNP@PCN@Gel exhibited analogous NO yields of 15.2 μM as the equivalent concentration of SNP (15.9 μM), higher that of Arg-PCN@Gel [31] and AI-MPDA nanoparticles [34]. NO-driven ONOO^−^ generation was confirmed by using L-tyr as a fluorescence probe in the presence of CO_2_ [27]. L-tyr would be oxidated into the dimerization in the presence of ONOO^−^. The excitation wavelength and emission wavelength of L-tyr dimerization was 313 and 406 nm, respectively. As shown in Fig. 4G, under light irradiation the ONOO^−^ generated by SNP@PCN@Gel was 2 and 4 times that of SNP@PCN and SNP@PCN@Gel in the dark, respectively, while the generation of ONOO^−^ by SNP and PCN-224 alone was negligible, indicating that light-triggered ONOO^−^ generation from SNP@PCN@Gel as evidenced by the dimerization of L-tyr [27].

Ag^+^ release is a prerequisite for the antibacterial property. Thus, Ag^+^ release amount in acetate buffer (pH5.0) and PBS (pH7.4) was monitored by ICP-MS, respectively. As shown in Fig. 4H, the release of Ag^+^ was fast in acetate buffer, and about 57% of Ag^+^ was released within 12 h, higher than that of 24% in PBS buffer. The slow release of Ag^+^ in PBS might be ascribed to the fact that a part of released Ag^+^ precipitated due to the lower solubility of AgCl (*K*_*sp*_ = 1.6 × 10^–10^) or Ag_3_PO_4_ (*K*_*sp*_ = 1.8 × 10^–8^) than that of AgAc (*K*_*sp*_ = 2.3 × 10^–3^) [44]. However, after that, both releases reached a plateau, likely due to the presence of Na^+^ or/and K^+^ in acetate or PBS buffer which affected the dissolution equilibrium of the hydrogels [44]. Our data confirmed the on-demand Ag^+^ release characteristic of SNP@PCN@Gel in responsive to the acidic environment of infected wounds.

For its activity on bacterial membranes and iron-sulfur clusters of dehydratases in bacteria, the released Ag^+^ disrupt dehydrogenases in respiratory chain, resulting in premature electron leakage. Then these electrons react with O_2_ in the cytoplasm, leading to O_2_^−^ generation [69]. Previous report has demonstrated that O_2_^−^ on its own was generally poorly reactive and only could attack a few biological molecules, and dismutation of O_2_^−^ would generate poorly toxic H_2_O_2_ [70]. However, the reaction between H_2_O_2_ and Fe^2+^ ions (referred to as Fenton reaction) can generate •OH, one of the strongest oxidants in nature, which cause serious damage to biomolecules. While SNP significantly induced O_2_^−^ and H_2_O_2_ generation, and extracellular Fenton reaction between H_2_O_2_ and Fe^2+^ released from SNP produced •OH to dominate the cytotoxicity [70]. Infectious microenvironment (IME) was often characterized by the peculiarities of acidic micromilieu and elevated expression of H_2_O_2_ [39, 40], being good for triggering Fenton reaction for enhanced CDT. The •OH-producing experiments verified that SNP@PCN@Gel with a higher fluorescence intensity at 446 nm could effectively produce •OH (Fig. 4I), and the absorption peak intensity significantly increased with the increase in the temperature from 25 °C to 62 °C (Fig. 4J), demonstrating that photothermal effect enhanced CDT by temperature-accelerated •OH generation. Finally, the intracellular ROS and NO was characterized by using DCFH-DA and DAF-FM as indicator [51, 52], and photographed by the fluorescence microscope. Compared to the control group, SNP@PCN@Gel concurrently generate ROS and NO with exposure to laser irradiation (Fig. 4K and L), and the corresponding fluorescence intensities were shown in Fig. 4M and N, in consistent with the previously reported data [51]. Based on our findings, we reasonably presumed that SNP@PCN@Gel hydrogels could potentially serve as photoactivatable “all-in-one” therapeutic hydrogels for synergistic PTT, PDT, CDT, IT and GT therapy and highly efficient photo-responsive NO carrier.

### In vitro antibacterial activity

**Fig. 5:**
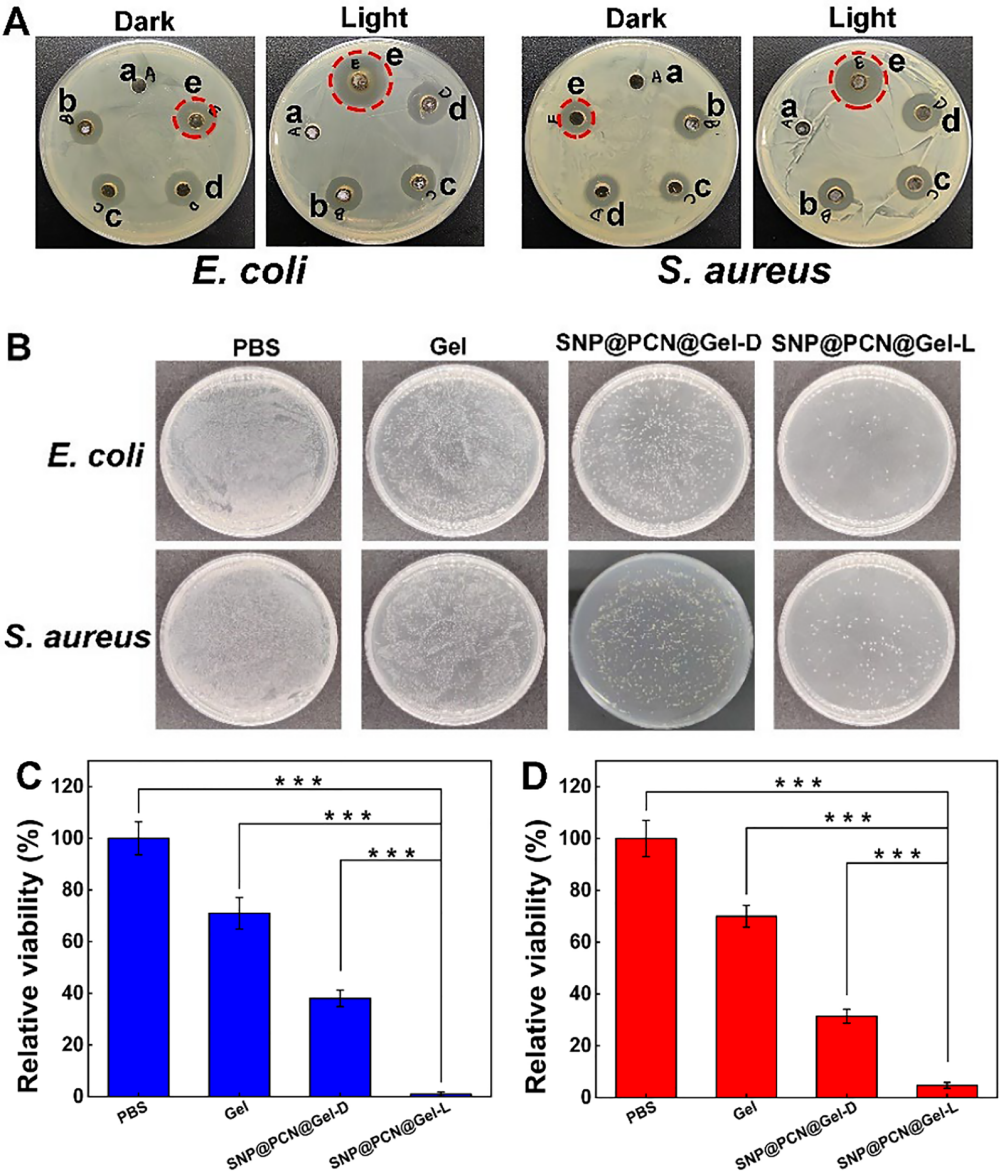

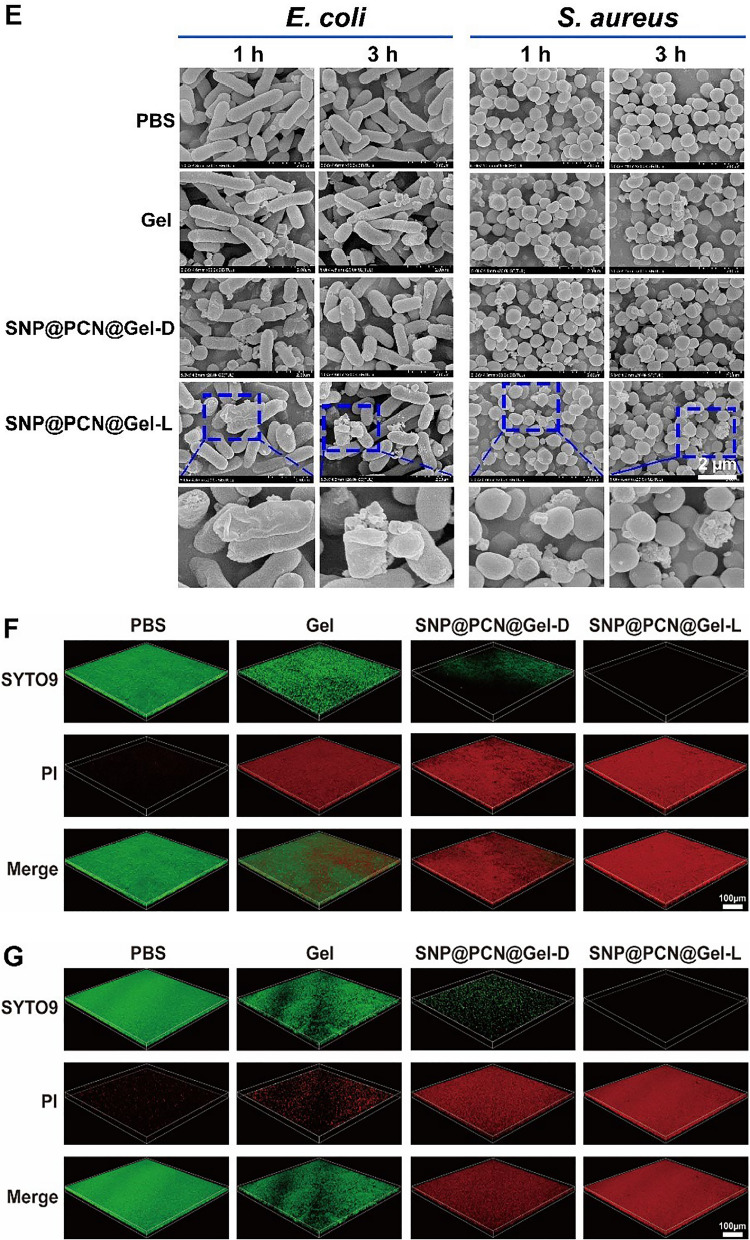
In vitro antibacterial activity. **A** Antibacterial zone inhibition images for various treatments. a PBS, b Gel, c PCN@Gel, d SNP@Gel, e SNP@PCN@Gel. **B** Photographs of *E. coli* and *S. aureus* agar plates. **C** and **D** The relative viability of *E. coli* and *S. aureus* after various treatments. **E** SEM images of *E. coli* and *S. aureus* after various treatments. **F** and **G** Living and dead biofilms of *E. coli* and *S. aureus* after various treatments, respectively. ***p < 0.001

Effectively controlling bacterial infection is one of the key requirements for wound healing. Based on the light-responsive synergistic therapy capability, the in vitro antibacterial activity against Gram-positive (*E. coli*) and Gram-negative (*S. aureus*) bacteria was firstly tested by the Oxford cup method and the microplate assay [45]. As expected, the PBS group did not exhibit any antibacterial activity, while the inhibitory rings of SNP@PCN@Gel were obviously observed even in the dark, maybe ascribing to the antibacterial effect of Ag^+^ and CMCS (Fig. 5A). However, upon exposure to light irradiation the inhibition zones reached highly at 22 ± 0.76 mm and 18 ± 0.35 mm for *E. coli* and *S. aureus* respectively. *E. coli* was more susceptible to SNP@PCN@Gel plus laser treatment. Subsequently, the antibacterial ability was quantitatively evaluated by counting the number of living bacteria after different treatment. As shown in the representative photos (Fig. 5B), more than 95% of *E. coli* and most of *S. aureus* cells were eradicated by SNP@PCN@Gel plus light irradiation, in consistent with the analysis results of relative viability (Fig. 5C and D). To trace the bactericidal mechanism of SNP@PCN@Gel, SEM analysis was conducted to visualize directly the bacterial morphologies. As shown in Fig. 5E, E*. coli* and *S. aureus* in the control groups exhibited normal shapes with glabrous and integrated cytomembranes. The surface of *E. coli* treated with the blank Gel became slight rough and wrinkled, while *S. aureus* still kept relatively intact cytomembrane. With the assistant of photothermal performance, the bacteria cells treated with SNP@PCN@Gel lost their cellular integrity and were even completely collapsed. Especially, spillage of intracellular components from *E. coli* cells could clearly be observed due to the more severe destruction of cell wall and membrane, while *S. aureus* cells also presented obviously rough, wrinkled and were ruptured, indicating that light irradiation was essential to improve the killing efficiency of SNP@PCN@Gel.

Healing biofilm infections is often difficult due to the specific microbial community structure which prevents the antimicrobial agent penetration. Therefore, we explored if SNP@PCN@Gel penetrated and thus eradicated the mature biofilms. SYTO9 can enter living bacteria with intact cell membranes and display green fluorescence, whereas PI only can enter bacteria with damaged membranes and show red fluorescence. Biofilms were incubated with SNP@PCN@Gel at 37 °C for 2 h, and the survival of bacteria was visually determined by green or red coloration (Fig. 5F and G). SNP@PCN@Gel-treated bacteria appeared entirely red under irradiation, indicative of severe disruption to the mature biofilms, consistent with SEM (Fig. 5E). In a word, all of these experiments in vitro testified that our designed SNP@PCN@Gel could attach to the microbe surfaces and subsequently produce a large amount of ROS such as OH and ONOO^−^ in situ, which killed the bacteria more directly and effectively.

### In vitro evaluation of cytocompatibility and hemocompatibility

**Fig. 6:**
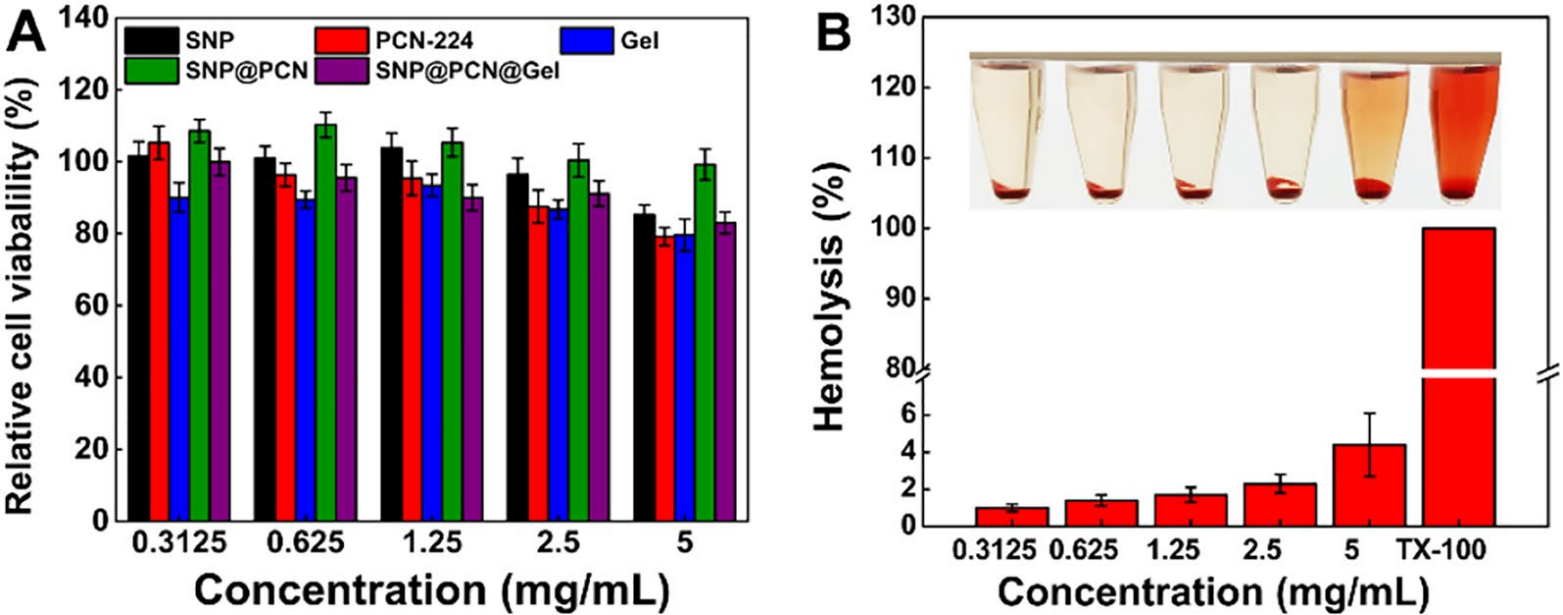
**A** Cell viability of HUVECs analysis by a standard MTT method. **B** Hemolytic assay of SNP@PCN@Gel by balb/c mice’s red blood cells

To assess the clinical application potential, cytotoxicity of SNP@PCN@Gel was evaluated by MTT assay against HUVECs in vitro. As shown in Fig. 6A, no significant cytotoxicity was observed in response to SNP@PCN@Gel treatment for 24 h, with cell viabilities higher than 80% even at a very high concentration (5 mg/mL), reflecting desirable cytocompatibility of SNP@PCN@Gel, due to the inherent biocompatibility of natural polysaccharide. Remarkably, for SNP@PCN group, the cell viability was higher than 100%, maybe ascribing to the presence of Zr^4+^, which promoted the proliferation of osteoblasts and fibroblasts, and facilitated tissue healing [31]. The blood compatibility was further evaluated by hemolytic tests. As depicted in Fig. 6B, SNP@PCN@Gel showed negligible hemolytic effect (< the national standard of 5%) even up to 5 mg/mL, exhibiting excellent hemocompatibility. Taken together, excellent cytocompatibility and hemocompatibility provided reliable evidence for further in vivo testing and future biomedical applications.

### Evaluation of pro-angiogenic effects in vitro

**Fig. 7:**
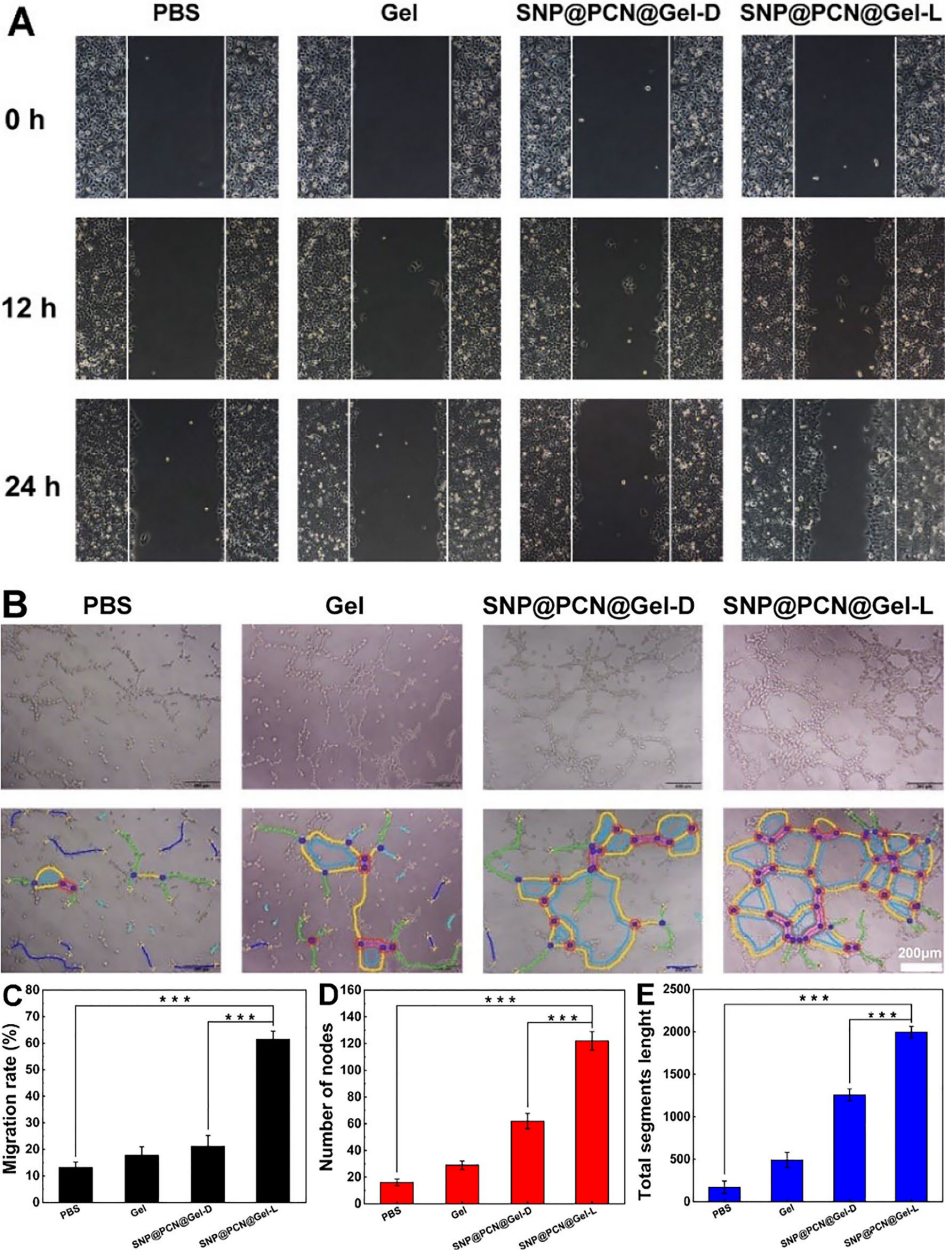
**A** Digital images of HUVECs after treatment with PBS, Gel, and SNP@PCN@Gel for 0, 12, and 24 h. **B** Images of tube formation after different treatments. **C** HUVECs migration rate after different treatments. **D** and **E** Number of nodes and total segments length in tube formation assay. ***p < 0.001

The cell migration is favor of wound healing, therefore, the effect of SNP@PCN@Gel on the migration ability of HUVECs was investigated by 2D scratch assay. HUVECs were cultured in a 6-well plate and then scratched using a pipette tip to create a wound. As presented in Fig. 7A, obvious cell migration was observed in the scratch area after 24 h of incubation. Comparatively, the migration rate of the SNP@PCN@Gel-L group was 61.5%, significantly higher than those in other groups (Fig. 7C), indicating SNP@PCN@Gel-L could effectively promote cell migration. Next, the ability of SNP@PCN@Gel-L to induce the tube formation was assessed. Compared with PBS group, a considerable number of capillary-like structures were observed in SNP@PCN@Gel-L group after 4 h of incubation (Fig. 7B), with an obvious increase in nodes and vascular sprouts, and longer tubules (Fig. 7D and E), mainly due to the presence of light-triggered NO generation. It was reported that NO regulated the stability HIF-1α protein which was the master regulator of angiogenesis in wound sites [38, 71], in turn facilitating the angiogenesis and endothelialization of wounds [38, 72]. These results indicated that SNP@PCN@Gel could be used as an efficient NO generator, promoting vascular endothelial cell migration and angiogenesis, all of which accelerated wound healing.

### In vivo infected-wound regeneration by SNP@PCN@Gel

**Fig. 8:**
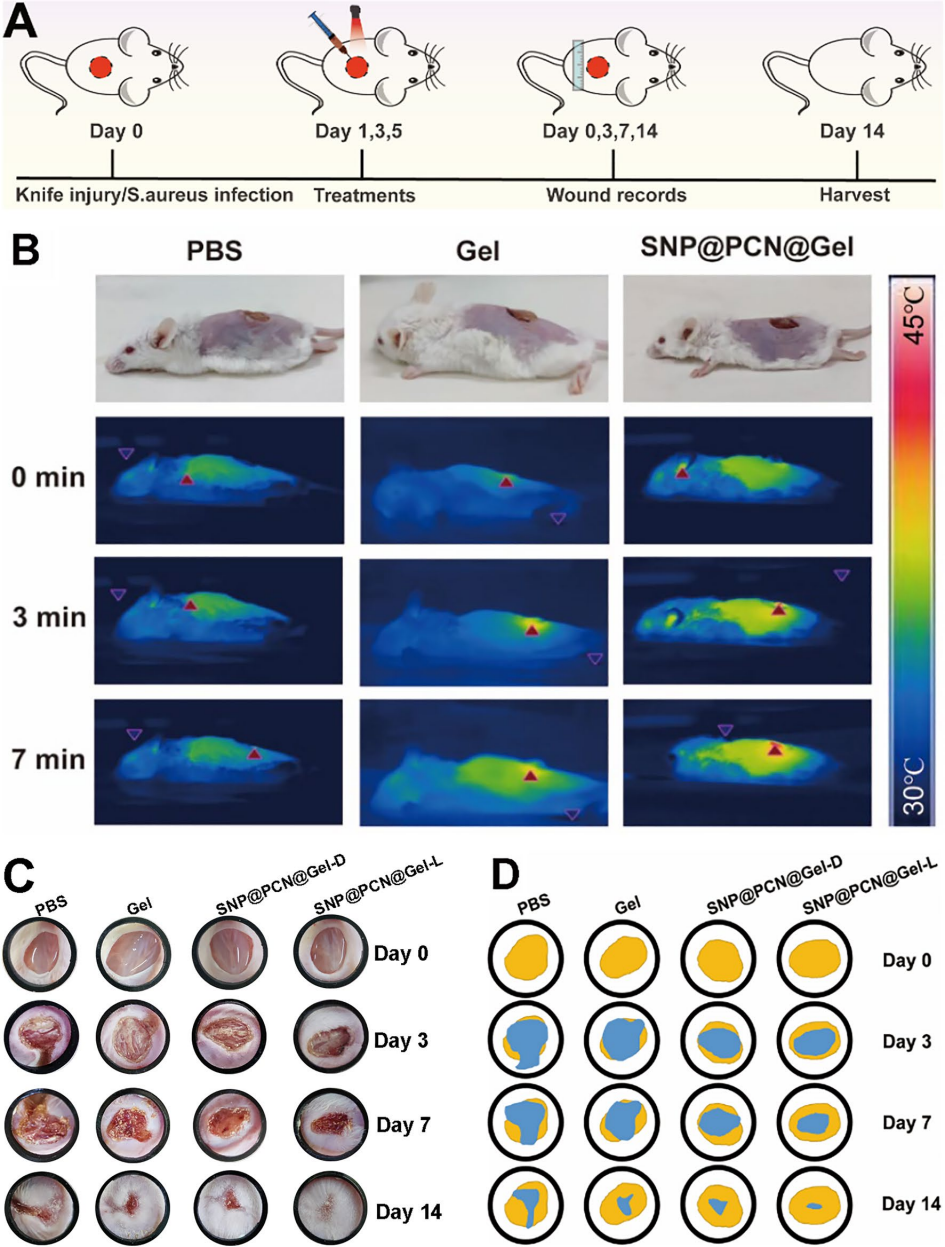

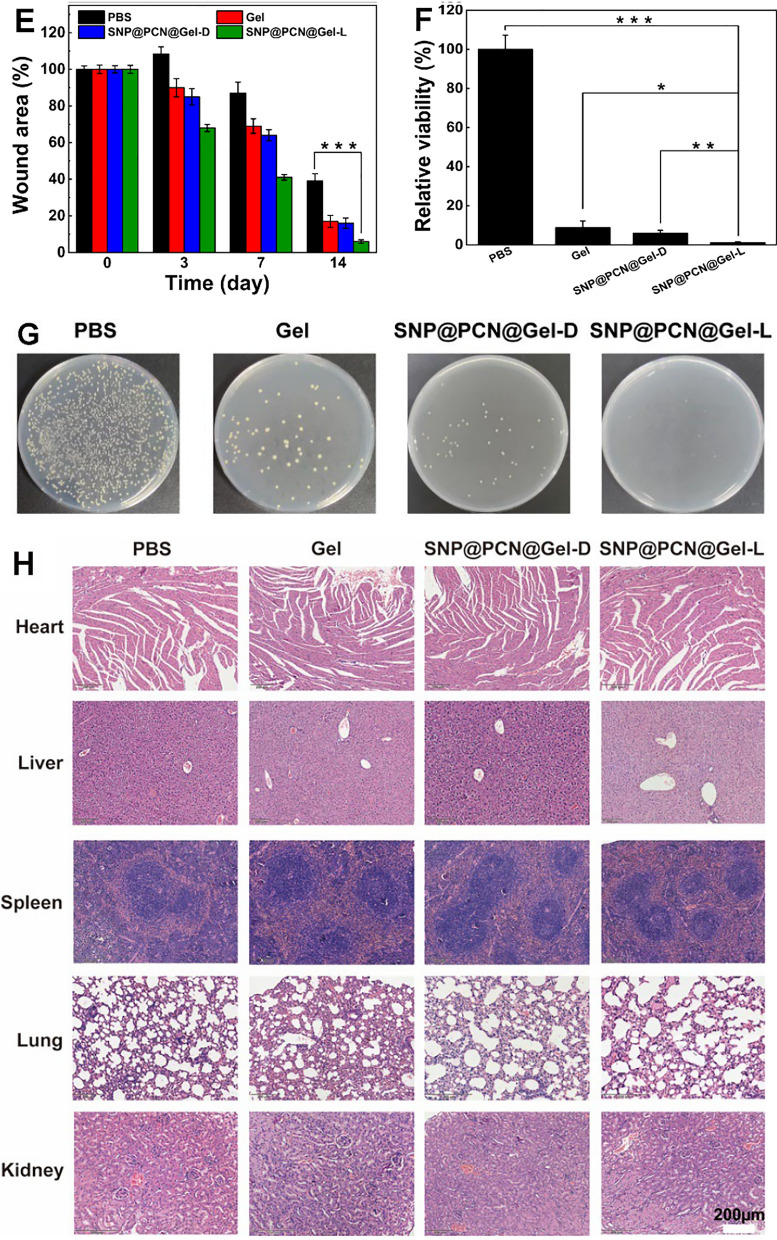

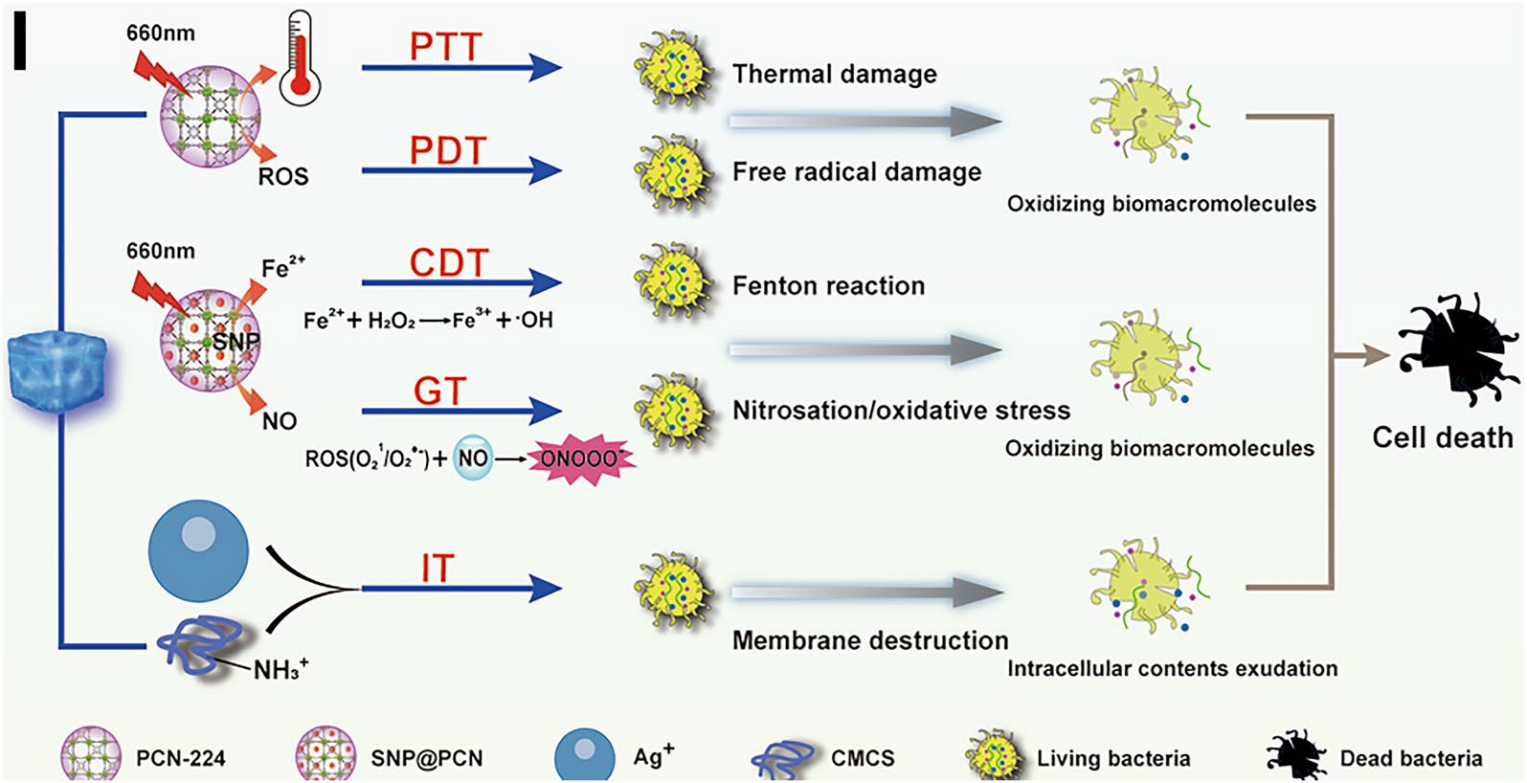
The therapeutic effect of SNP@PCN@Gel on infected skin wound in vivo. **A** Schematic illustrating the treatment regime. **B** Photothermal assay. **C** Representative photographs of wound tissues after different treatments. **D** Wound healing boundaries in different groups. **E** Quantification of the wound closure rate. **F** Relative bacterial survival rates. **G** The bacteria collected from wound tissue after various treatments were cultured on agar plates on days14. **H** H&E staining images of the mice’ major organs. **I** The prospective antibacterial mechanism of SNP@ PCN@Gel caused by synergistic effects of PTT, PDT, GT, CDT, and IT therapy. ***p < 0.001

Inspired by the observed excellent antibacterial activity and biocompatibility in vitro, we further evaluated the potential of our designed antimicrobial hydrogels for resisting wound infection in vivo according to the established treatment procedures (Fig. 8A). Firstly, balb/c mice with 1 cm diameter wound on the back were employed and 100 μL *S. aureus* (1.0 × 10^7^ CFU/mL) were inoculated to fabricate bacteria-infected wound. Mice were then randomly separated into four different groups (n = 6): treated with (I) PBS, (II) Gel, (III) SNP@PCN@Gel-D, and (V) SNP@PCN@Gel-L, respectively. SNP@PCN@Gel-L groups were exposed to the laser irradiation on days 1, 3, and 5 for 10 min, respectively. During the therapeutic process, wound area bacterial on days 0, 3, 7, and 14 and burden was counted on day 14, to evaluate the wound healing, and the death number of mouse was also recorded. As shown in Fig. 8B, the wound temperature of SNP@PCN@Gel treated mouse rose to 46.3 ± 1.6 ℃ within 7 min under light irradiation, which was significantly higher than that of PBS group (37.1 ± 0.88 ℃) and Gel group (38.9 ± 1.3 ℃), indicating the realization of photothermal therapy. The macroscopic process of wound healing for each group was monitored at preset time intervals (0, 3, 7, and 14 days) using a digital camera (Fig. 8C). After 3 days of treatment, the wounds of control group displayed and inflammation and some yellow festering, the same phenomenon was also seen in the wounds with attached blank Gel, mainly due to bacterial infections. Despite less serious, the wounds of mice in SNP@PCN@Gel-D group also appeared abscesses and inflammation to varying degrees. Notably, the wounds with the attached SNP@PCN@Gel exhibited no edema or festering under laser irradiation. Compared with other treatment groups, the wound area size in SNP@PCN@Gel-L group was significantly smaller on days 7. The wounds with the attached SNP@PCN@Gel were nearly healed and new epidermal tissue appeared under laser irradiation after 14 days of therapy, whereas the wounds in the other treatment groups exhibited showed irregular scars, demonstrating SNP@PCN@Gel plus light irradiation not only availably hindered the wound infection but also prominently accelerated wound healing process. Additionally, based on the wound photographs the wound area was quantitatively measured (Fig. 8D) to estimate the wound healing rate. As shown in Fig. 8E, the SNP@PCN@Gel-L group exhibited a more brilliant wound healing rate in comparison with other treatment groups. After 7 days of therapy, the wound area of PBS group was 87%, while it reduced to 41% in SNP@PCN@Gel-L group. As expected, the average wound area of SNP@PCN@Gel-L group had shrunk to 6%, significantly lower than that of the PBS group (39%) after 14 days. The results demonstrated that *S. aureus* infection could handicap wound regeneration. To evaluate the bactericidal effect, the numbers of bacteria were quantified by harvesting the wound tissues after 14 days of treatment. As depicted in Fig. 8F and G, the control group of colony counts showed plenty of bacteria, suggesting that these mice still suffered severe bacterial infections. In contrast, the grown colonies in SNP@PCN@Gel-L group were significantly lower, and revealed that SNP@PCN@Gel-L caused the most effective bactericidal effect. Whether SNP@PCN@Gel treatment produced in vivo side effects was further evaluated by analyzing body weight and major internal organ sections of mice in various treatment groups. It was found that no significant difference was observed in the mice body weight of each group during the whole treatment period (Fig. S10), suggesting that SNP@PCN@Gel has no obvious biological toxicity. As except, no appreciable histological abnormalities or the occurrence of side reactions in any organ (liver, heart, lung, spleen, and kidney) were observed in the SNP@PCN@Gel group even after 14 days of therapy (Fig. 8H), further verifying the good biosafety in vivo of SNP@PCN@Gel.

Based on the above-mentioned results, a conceivable mechanism for bactericidal performance of SNP@PCN@Gel was illustrated in Fig. 8I. Upon SNP@PCN@Gel attached onto the skin wound, five successive events contributed to the great bacterial inhibition efficiency of SNP@PCN@Gel under light irradiation. (i): PCN-224 converted light to heat, leading to local hyperthermia which killed bacteria by bacterial protein denaturation and irreversible bacterial destruction [29, 30] (Implementing PTT). (ii): the released Ag^+^ and protonated NH_3_^+^ in CMCS tightly interacted with the negatively charged bacterial membrane, which improved the membrane permeability thus resulting in bacteria lysis and the intracellular contents exudation [73], and increased the contact between bacteria and other bioactive components (e.g., SNP@PCN nanoparticles, later released Ag^+^), consequently facilitating the penetration of them into the bacteria (Implementing IT). (iii): PCN-224 converted light to ROS, which further killed bacteria by disrupting bacterial cell membranes and DNA [28] (Implementing PDT). (iv): the released NO from SNP reacted with ROS to generate ONOO^−^ which exacerbated the total damage to the bacterial cell by triggering free radical peroxidation [31, 35–37], and accelerated intracellular GSH consumption consequently enhancing the efficiency of PDT (Implementing GT and enhanced-PDT). (v): the released Fe^2+^ reacted with H_2_O_2_ in infectious microenvironment [39, 40] and Ag^+^-induced generated H_2_O_2_ [69, 70] to produce •OH to induce cell death (Implementing CDT). These results confirmed that SNP@PCN@Gel plus light irradiation exhibited synergetic antibacterial effect with negligible biotoxicity, and could be potentially employed to treat infected skin wounds and accelerate the tissue regeneration.

**Fig. 9:**
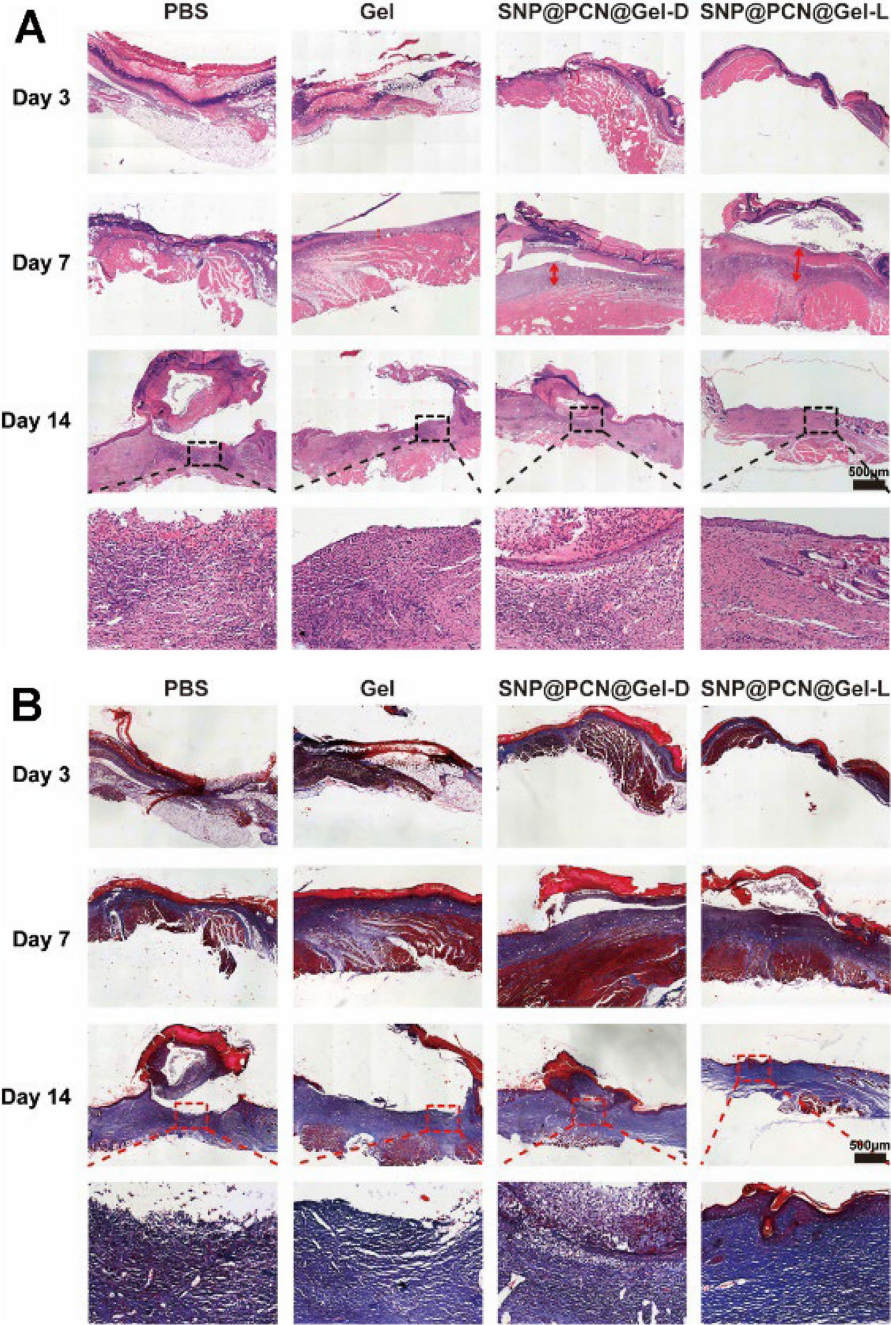

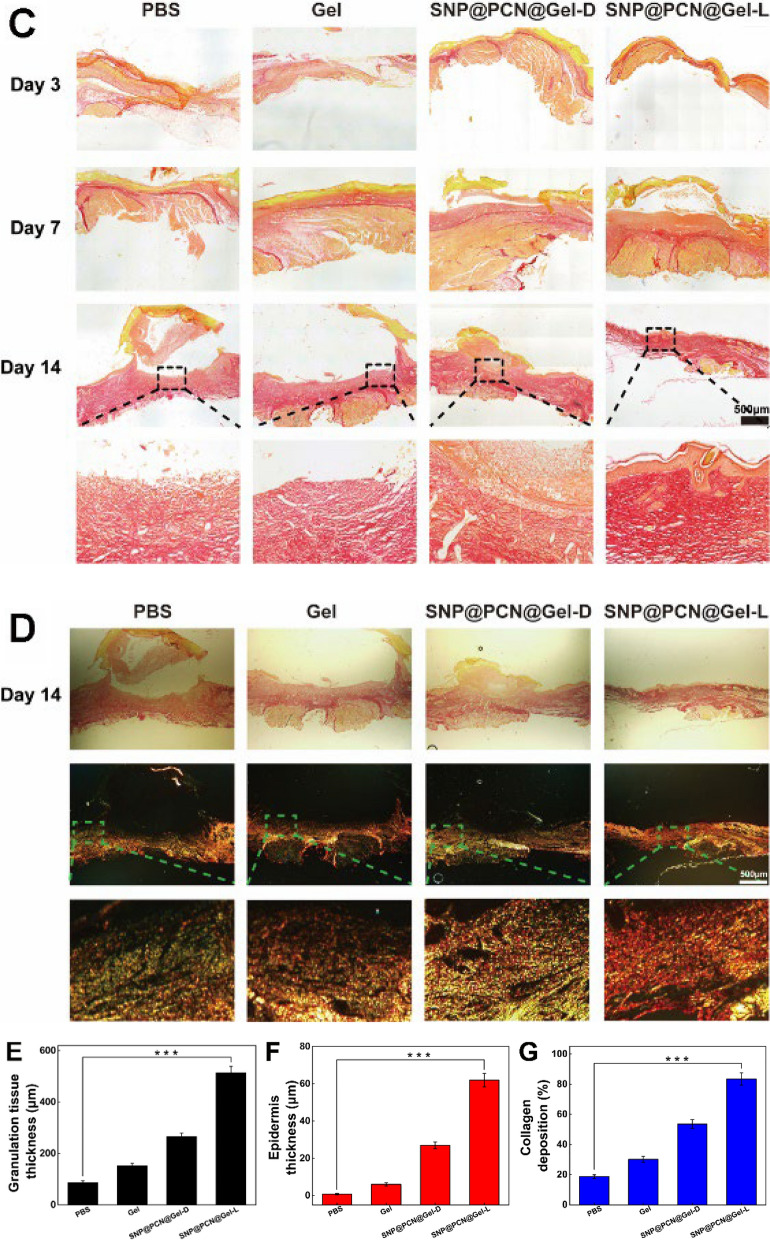
**A** H&E, **B** Masson and **C** Sirius Red staining of the new growing skin tissues. **D** Cross-polarized optical microscope images of birefringent collagen on 14 days. Collagen type was determined by the fiber thickness, with collagen type I appearing as large orange-red fibers and collagen type III appearing as thin green filamentous fibers. **E** Granulation thickness, **F** Epithelial thickness, **G** Collagen deposition. ***p < 0.001

Histological analysis was carried out on days 3, 7 and 14 to evaluate the wound healing efficacy by semi-quantitative analysis on the regeneration of the defective epidermis. Firstly, H&E staining was employed to observe the epithelialization degree. On days 3, each group exhibited visible new granulation tissue and proliferation of fibroblasts (Fig. 9A). With the time prolong after treatment, neoplastic granulation tissue became thicker at 7th day (Fig. 9E), and the SNP@PCN@Gel-L group displayed longer migration distances and neovascularization, and the border between the surrounding normal and neonatal epidermis was not obvious, suggesting a better wound healing outcoming. On days 14, for SNP@PCN@Gel-L group, the epidermis became thicker and was almost completely healed (Fig. 9F), and the tightly connected connective tissue was subcutaneously visible. In contrast, inflammatory cells still appeared in PBS group, implying that the epidermis was not completely healed. Then Masson’s trichrome staining was carried out to observe the formation and deposition of collagen (Fig. 9B and G). On days 3, all group presented sparsely distributed collagen deposition. On days 7 and 14, Masson’s staining of the whole dermal trabeculae in the SNP@PCN@Gel-L group became bluer, implying denser granulation tissue deposition and better collagen bundles. Smaller stained areas and less fragmented collagen bundles were observed in other groups. It was reported that the collagen type I and III were closely related to the skin wound healing process and the quality of repair [72, 74]. A higher ratio of collagen I to III in skin wound healing suggested collagen maturation and enhanced mechanical strength of the healing wound tissue, being good for the wound more resistant to injury [75]. As shown in Fig. 9C and D, on days 14, for SNP@PCN@Gel-L group, the mature collagen type I was predominant in the collagen, while the majority of collagen was immature collagen type III in other groups. The results demonstrated that SNP@PCN@Gel-L not only raised the amount of collagen during the skin wound healing but also promoted the collagen maturation, consequently accelerating skin wound healing.

**Fig. 10:**
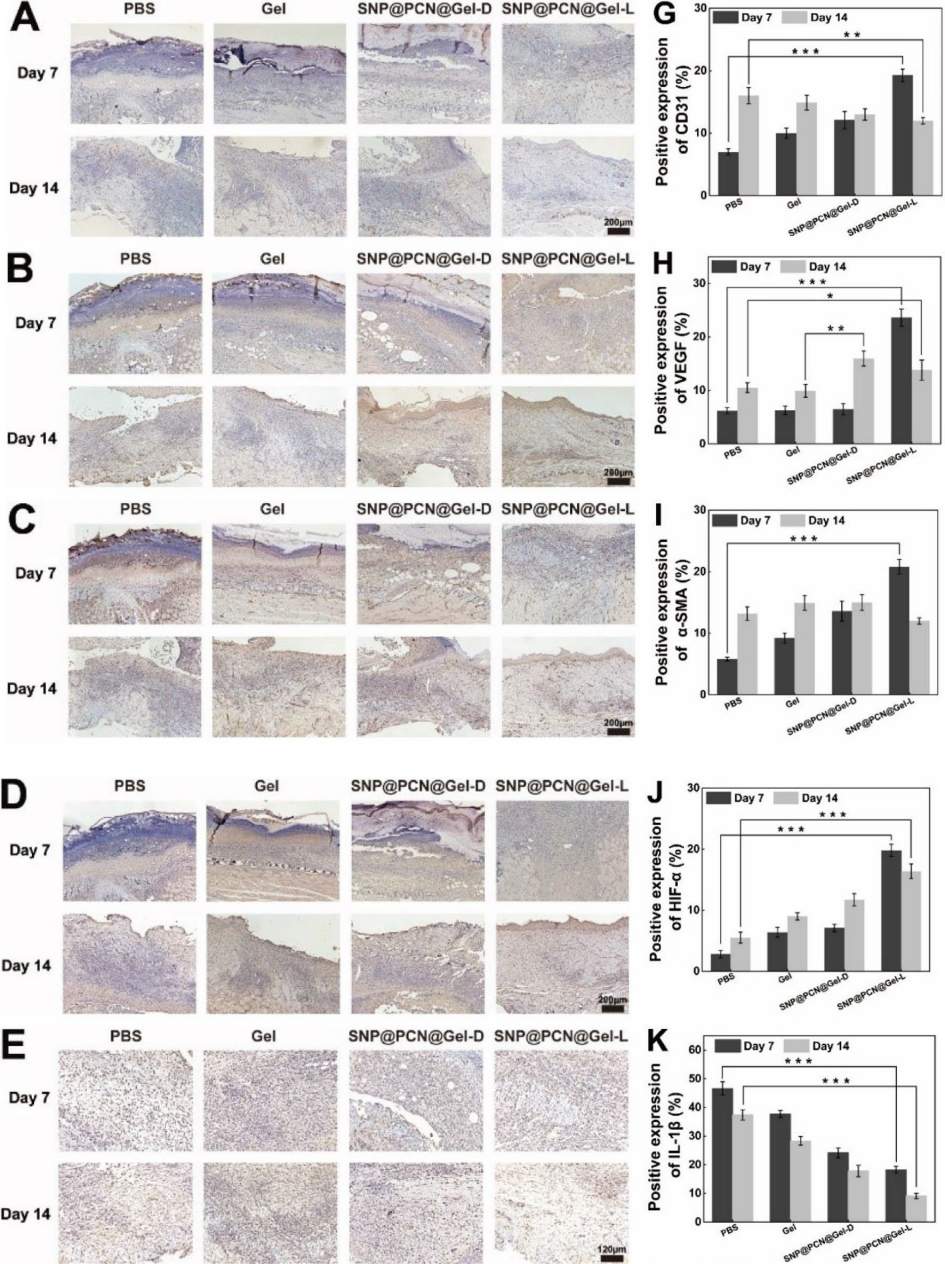

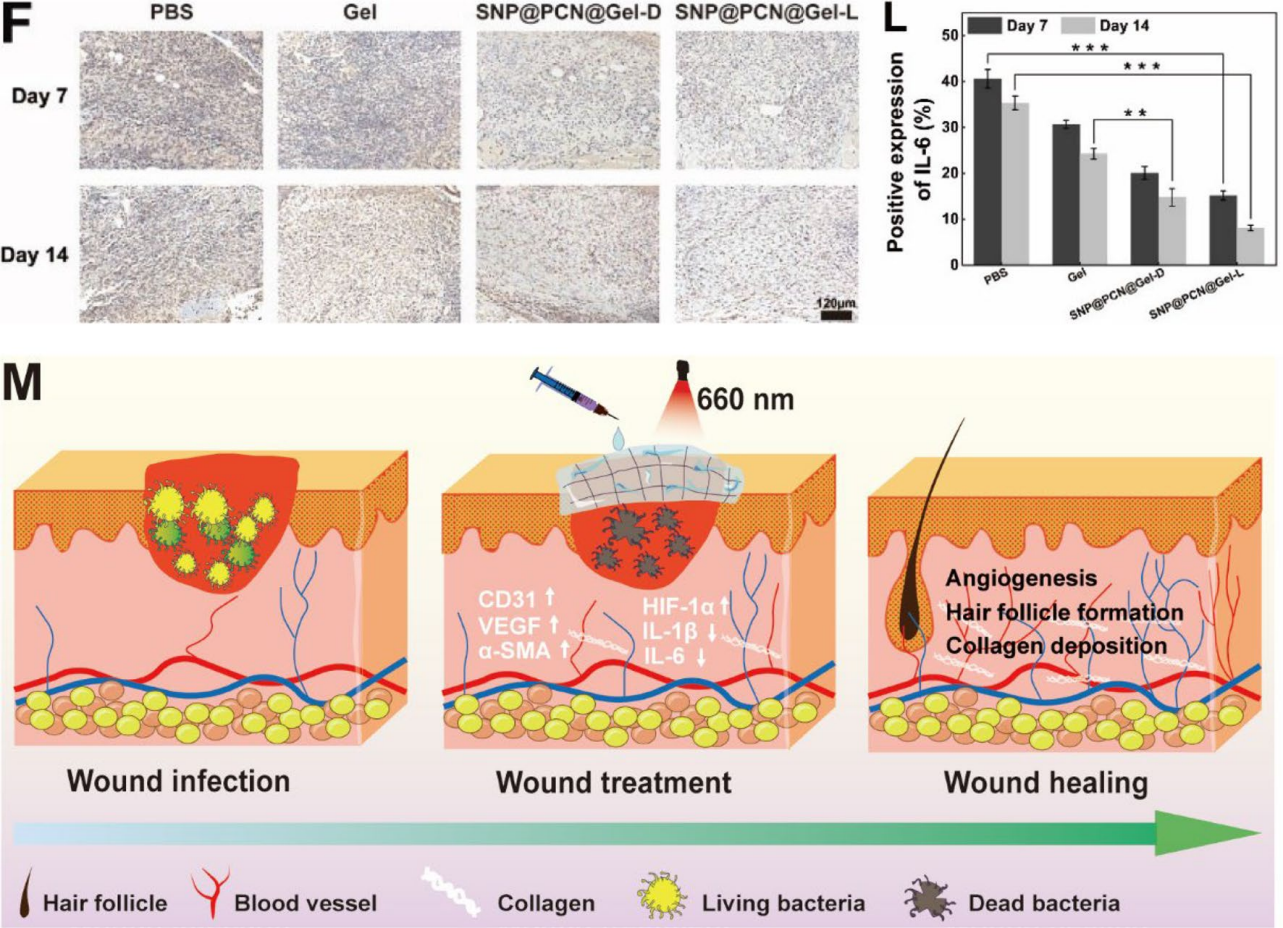
IHC analysis of the wound tissues on days 7 and 14 post injury. **A** CD31staining of skin tissue sections, **B** VEGF staining of skin tissue sections, **C** α-SMA staining of skin tissue sections, **D** HIF-1α staining of skin tissue sections, **E** IL-1β staining of skin tissue sections, **F** IL-6 staining of skin tissue sections; **G** CD31 expression, **H** VEGF expression, **I** α-SMA expression, **J** HIF-1α expression, **K** IL-1 βexpression, **L** IL-6 expression. **M** Schematic illustrating wound healing mechanism. *p < 0.1, **p < 0.01, ***p < 0.001

Angiogenesis is the primary concern for the wound healing thanks to the blood vessels provide nutrition to cells, a phenomenon which is related to regeneration and granulation [38]. The effect of various treatments on the neovascularization was assessed by IHC staining of CD31 (a specific vascular system marker), VEGF (a specific neovascularization marker), and α-SMA (a specific scar marker) on days 3 (Fig. S11A-C), 7 and 14. As shown in Fig. 10A–C, the expression level of CD31, VEGF and α-SMA, especially in the SNP@PCN@Gel-L group, were significantly higher than that of PBS group on days 7. The elevated expression of endovascular factors was conductive to angiogenesis and accelerated the early stage of wound healing. Importantly, Neovascularity recruited more growth factors [75], and provided nutrients and oxygen to promote cell growth and proliferation, essential for skin regeneration [76]. However, all expression level of CD31, VEGF and α-SMA in the SNP@PCN@Gel-L group decreased after 14 days in comparation with the control group (Fig. 10D–I), being good for promoting the later stage of scarless skin formation (remodeling stage) of wound healing [77]. It was reported that opportunely degeneration of the temporary built immature blood vessels was of significance for avoiding the formation of scar [78]. Especially, scar contraction in vivo has direct association with a-SMA expression level in scar tissue. Therefore, the timely and phased angiogenesis capability of SNP@PCN@Gel-L accelerated wound closure and promoted scarless wound healing by restraining excessive neovascularization and subsequent over collagen deposition in the remodeling phase [77]. Previous study has demonstrated that cell migration and VEGF secretion depended on the stabilization of HIF-1α, while NO could improve HIF-1α stabilization and increase HIF-1α protein levels by delaying its degradation [38]. Therefore, IHC staining was carried out to confirm the expression of HIF-1α. As shown in Fig. S11D and Fig. 10D and J, SNP@PCN@Gel-L group showed greater expression of HIF-1α than that of other groups. The results were in line with the in the above results (Fig. 10B and H). Besides angiogenesis, local inflammation also played key role in skin wound healing [79]. After 7 days and 14 days of treatment, IHC staining for interleukin-1β (IL-1β) and interleukin-6 (IL-6) was conducted to evaluate local inflammation during wound healing. The expression level of IL-1β (Fig. S11E, Fig. 10E and K) and IL-6 (Fig. S11F, Fig. 10F and L) were significantly lower in the SNP@PCN@Gel-L group than in other groups, verifying that SNP@PCN@Gel-L effectively alleviated the inflammatory response and promoted the wound healing. The accelerated wound healing might be ascribed to the following reasons (Fig. 10M): (i) CMCS-based hydrogels maintained the wound part moist, which contributed to the wound healing [1, 5, 6]. (ii) NO promoted wound healing by improving the stability of HIF-1α [38]. (iii) up-regulation expression of CD31, VEGF and α-SMA promoted neovascularity and accelerated the early stage of wound healing, while subsequent down-regulation promoted the remodeling stage of wound healing by restraining excessive neovascularization and collagen deposition [77]. (iv) HIF-1α increased VEGF secretion, further promoting neovascularity. (v) down-regulation expression of IL-1β and IL-6 alleviated the inflammatory response and further promoted the wound healing.

## Conclusion

In this study, a double metals-driven synergizing PTT, PDT, CDT, GT and IT CMCS-based hydrogels (SNP@PCN@Gel) for treating bacteria-infected skin wound was neatly fabricated: the hydrogels containing PCN-224 as a PDT platform for generating ROS, incorporating SNP in PCN-224 for producing NO to react with ROS to produce more lethal ONOO^−^ and enhance HIF-1α stabilization, releasing Ag^+^ and Fe^2+^ for driving Fenton reactions to generate •OH thereby significantly inducing bacteria cell death, and by CMCS and released Ag^+^ to damage bacteria cell membrane thus improving the membrane permeability. SNP@PCN@Gel showed interconnected and homogeneous porous structure, excellent self-healing capacity, low cytotoxicity, good blood compatibility, and robust antibacterial activity. More importantly, SNP@PCN@Gel significantly promoted reepithelization, the formation granulation tissue, collagen deposition and angiogenesis as well as wound contraction. To sum up, these factors synergistically contributed to facilitating infected skin wound closure. Therefore, this study provided a novel avenue to fabricate injectable self-healing hydrogels as safe and effective antibiotic alternatives for synergistic antibacterial applications with excellent biocompatibility and activity.

### Supplementary Information

Supplementary Materials 1. Table S1. Orthogonal test scheme for the investigation of hydrogels generation. Table S2. The detailed dosage of each component for the preparation of hydrogels. Fig. S1. The stability of PCN-224 and SNP@PCN nanoparticles. Fig. S2. The content of the elements of PCN-224 (A) and SNP@PCN nanoparticles (B). Fig. S3. SNP standard curve of quantitative determination. Fig. S4. Digital images of various hydrogels (test NO. 1-25). Fig. S5. SEM images of various hydrogels (test NO. 8, 9, 10, 15, 19). Fig. S6. Strain-sweep tests of number 8, 9, 10, 15 and 19 hydrogels. Fig. S7. Shear thinning tests of number 8, 9, 10, 15 and 19 hydrogels. Fig. S8. Time-sweep tests of number 8, 9, 10, 15 and 19 hydrogels. Fig. S9. (A) Standard curve of NO constructed using an NO detection kit. (B) Release of NO at the different time periods. Fig. S10. Body weight of Balb/c mice in different treatment groups at different time points. Fig. S11. (A) CD31, (B) VEGF, (C) α-SMA, (D) HIF-1α, (E) IL-1β and (F) IL-6 staining of skin tissue sections on day 3 post injury.

## Data Availability

The data are available from the corresponding author upon reasonable request.
