# Supplementary material for: Fast fabrication of “all-in-one” injectable hydrogels as antibiotic alternatives for enhanced bacterial inhibition and accelerating wound healing
Source: J Nanobiotechnology. 2024 Jul 26;22:439. doi: 10.1186/s12951-024-02657-4 (PMC11282694; doi:10.1186/s12951-024-02657-4)
Supplement: Supplementary file 1 — Supplementary Materials 1. Table S1. Orthogonal test scheme for the investigation of hydrogels generation. Table S2. The detailed dosage of each component for the preparation of hydrogels. Fig. S1. The stability of PCN-224 and SNP@PCN nanoparticles. Fig. S2. The content of the elements of PCN-224 (A) and SNP@PCN nanoparticles (B). Fig. S3. SNP standard curve of quantitative determination. Fig. S4. Digital images of various hydrogels (test NO. 1-25). Fig. S5. SEM images of various hydrogels (test NO. 8, 9, 10, 15, 19). Fig. S6. Strain-sweep tests of number 8, 9, 10, 15 and 19 hydrogels. Fig. S7. Shear thinning tests of number 8, 9, 10, 15 and 19 hydrogels. Fig. S8. Time-sweep tests of number 8, 9, 10, 15 and 19 hydrogels. Fig. S9. (A) Standard curve of NO constructed using an NO detection kit. (B) Release of NO at the different time periods. Fig. S10. Body weight of Balb/c mice in different treatment groups at different time points. Fig. S11. (A) CD31, (B) VEGF, (C) α-SMA, (D) HIF-1α, (E) IL-1β and (F) IL-6 staining of skin tissue sections on day 3 post injury. [file 12951_2024_2657_MOESM1_ESM.docx]

**Supporting Materials**

**Table S1.** Orthogonal test scheme for the investigation of hydrogels generation.

| Levels | CMCS (mg/mL) | Ag^＋^ (mol/L) | SNP@PCN (mg/mL) |
| --- | --- | --- | --- |
| 1  2  3  4  5 | 6.25  12.50  25.00  50.00  75.00 | 0.0  0.1  0.2  0.4  0.6 | 0.00  0.25  0.50  1.00  1.50 |

**Table S2.** The detailed dosage of each component for the preparation of hydrogels.

| Experimental number | CMCS (mg/mL) | Ag^＋^ (mol/L) | SNP@PCN (mg/mL) |
| --- | --- | --- | --- |
| 1  2  3  4  5  6  7  8  9  10  11  12  13  14  15  16  17  18  19  20  21  22  23  24  25 | 6.25  6.25  6.25  6.25  6.25  12.5  12.5  12.5  12.5  12.5  25  25  25  25  25  50  50  50  50  50  75  75  75  75  75 | 0  0.1  0.2  0.4  0.6  0  0.1  0.2  0.4  0.6  0  0.1  0.2  0.4  0.6  0  0.1  0.2  0.4  0.6  0  0.1  0.2  0.4  0.6 | 0  0.25  0.5  1.0  1.5  0.5  1.0  1.5  0  0.25  1.5  0  0.25  0.5  1.0  0.25  0.5  1.0  1.5  0  1.0  1.5  0  0.25  0.5 |

**
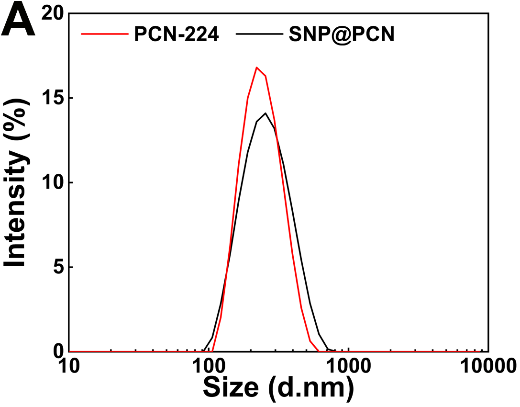

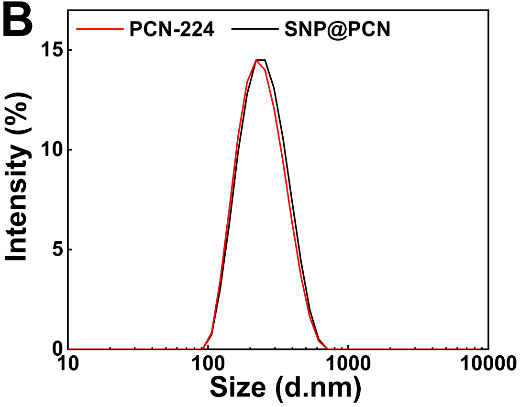
**

**
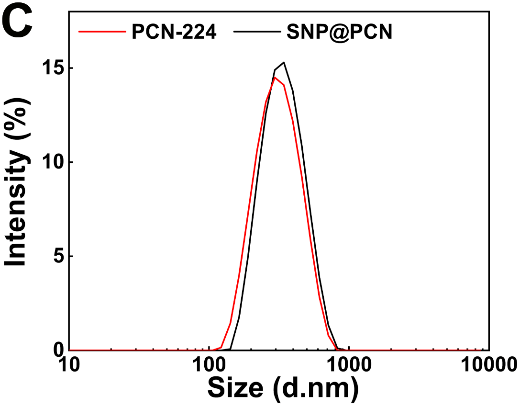
**

**Fig. S1.** The stability of PCN-224 and SNP@PCN nanoparticles. A) Size distribution of PCN-224 and SNP@PCN nanoparticles before and after loading SNP. B and C). Size distribution of PCN-224 and SNP@PCN nanoparticles after stored at room temperature for 7 and 14 days, respectively.


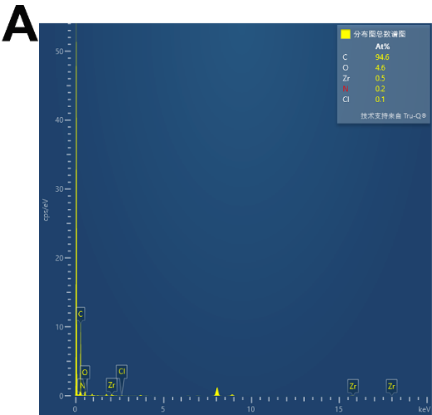

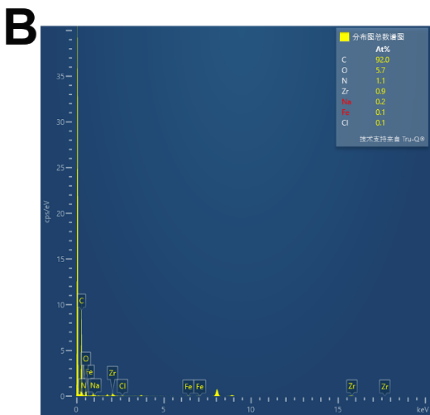


**Fig. S2.** The content of the elements of PCN-224 (A) and SNP@PCN nanoparticles (B).


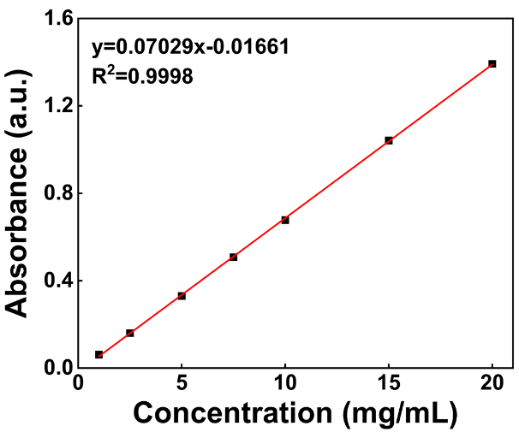


**Fig. S3.** SNP standard curve of quantitative determination.


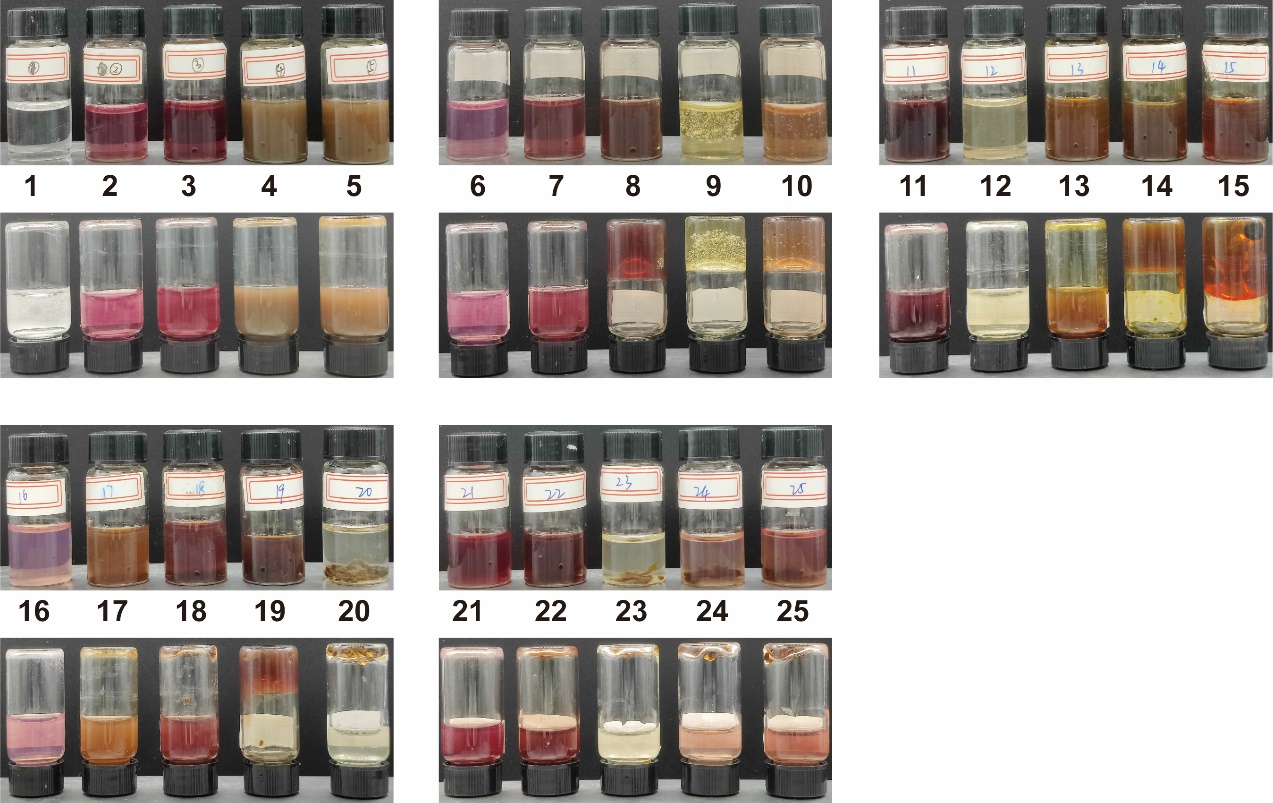


**Fig. S4.** Digital images of various hydrogels (test NO. 1-25).


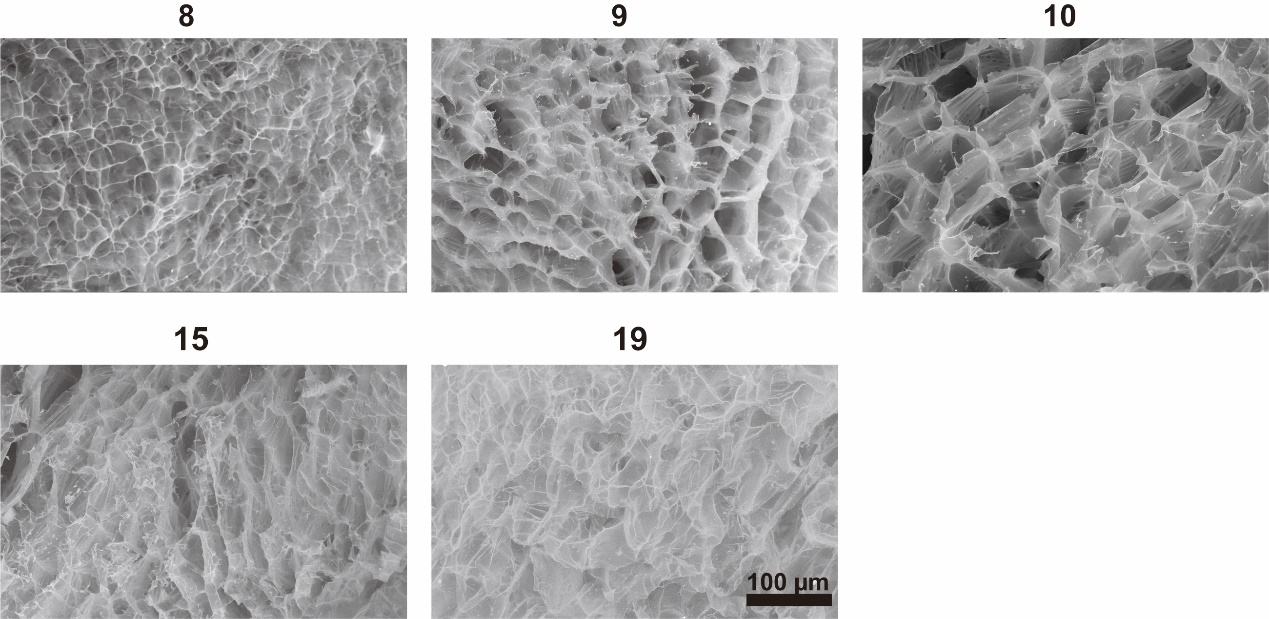


**Fig. S5.** SEM images of various hydrogels (test NO. 8, 9, 10, 15, 19).


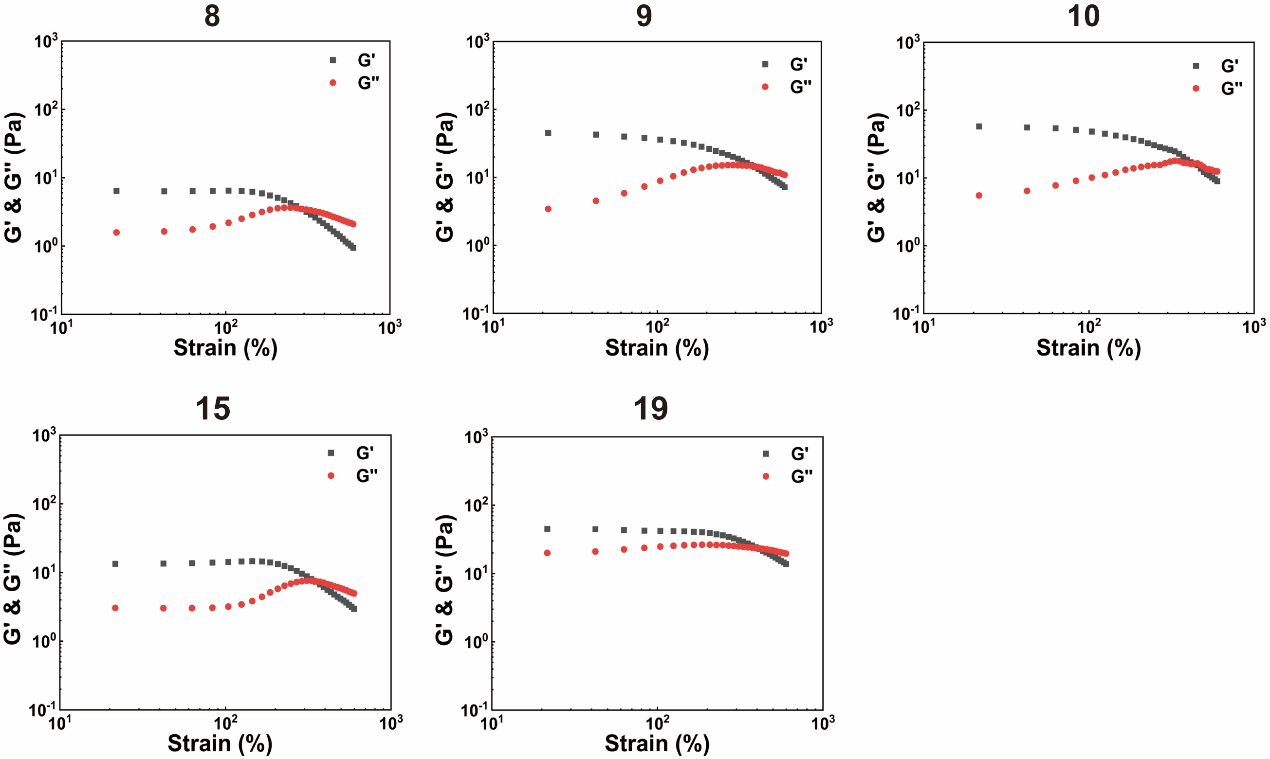


**Fig. S6.** Strain-sweep tests of number 8, 9, 10, 15 and 19 hydrogels.


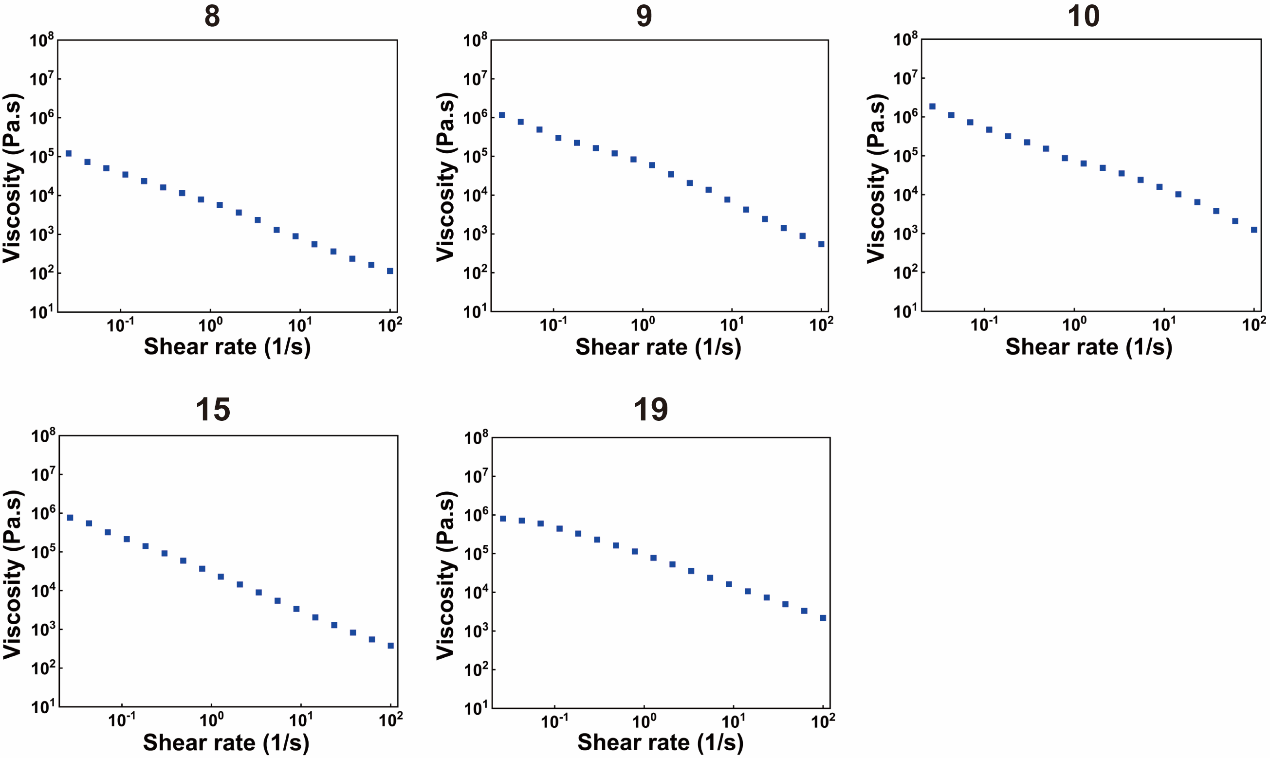


**Fig. S7.** Shear thinning tests of number 8, 9, 10, 15 and 19 hydrogels.


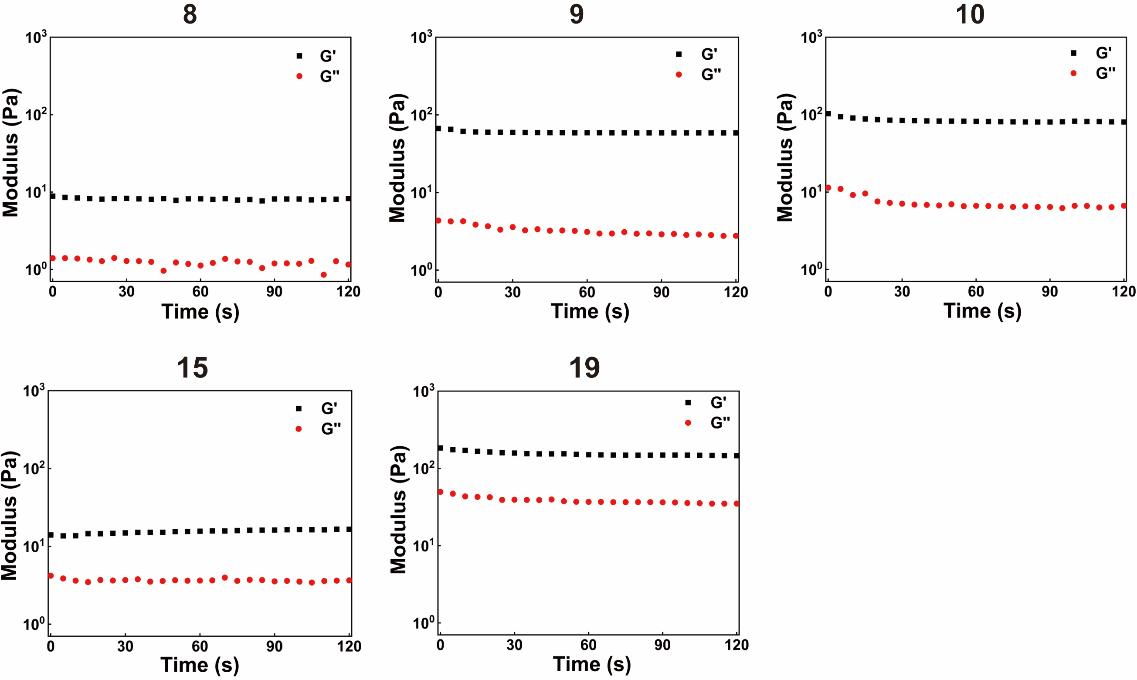


**Fig. S8.** Time-sweep tests of number 8, 9, 10, 15 and 19 hydrogels.


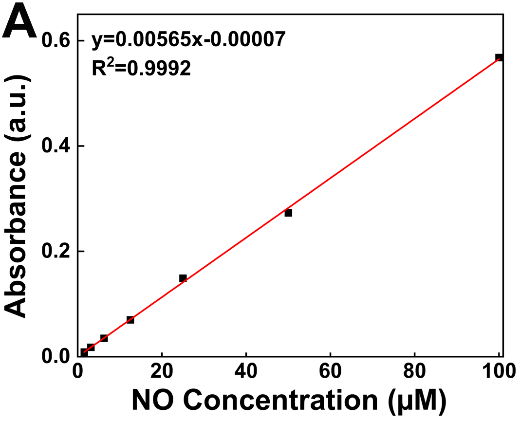

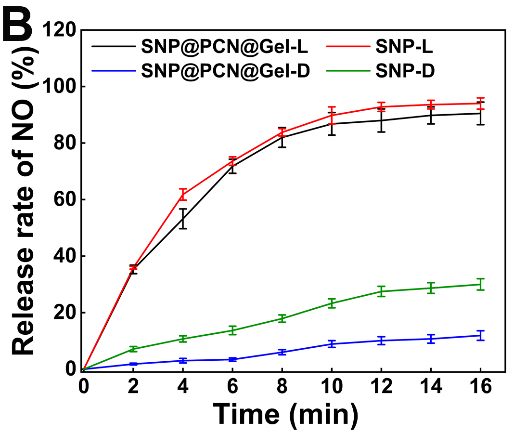


**Fig. S9.** (A) Standard curve of NO constructed using an NO detection kit. (B) Release of NO at the different time periods.


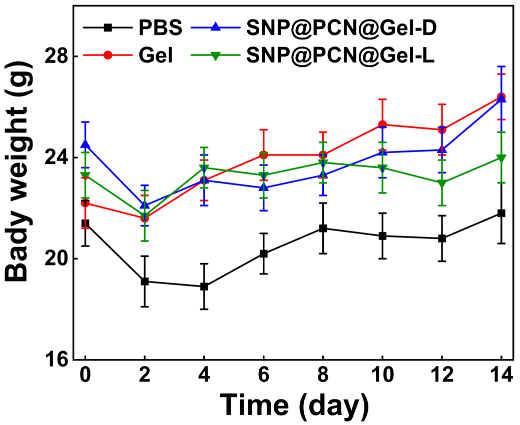


**Fig. S10.** Body weight of Balb/c mice in different treatment groups at different time points.


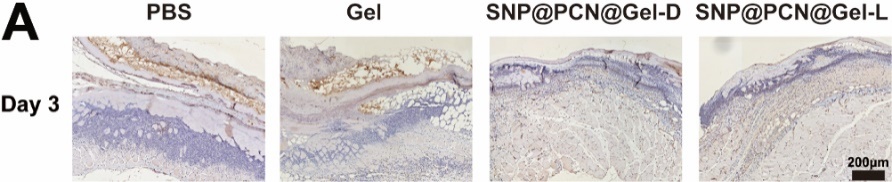


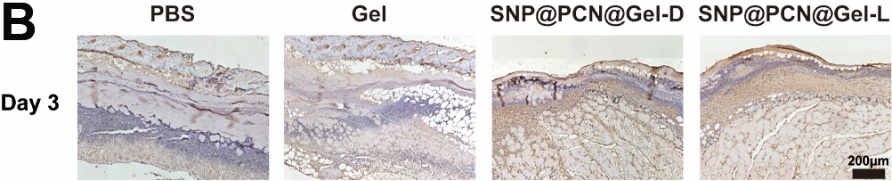


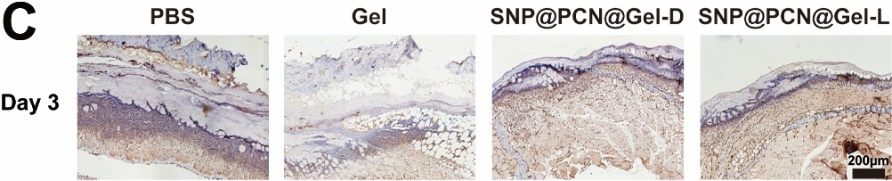


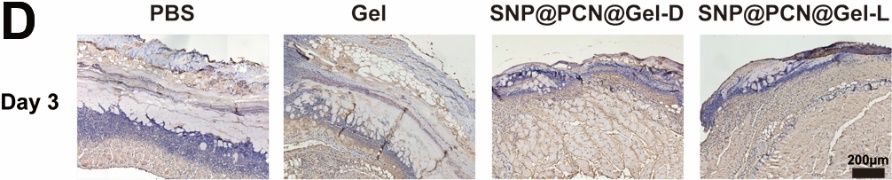


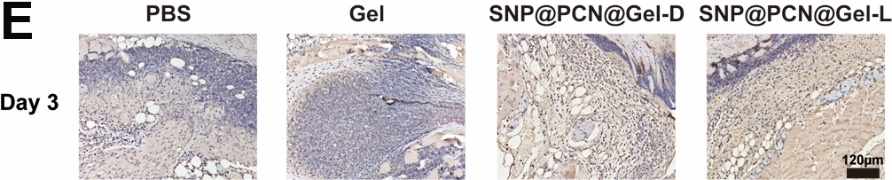


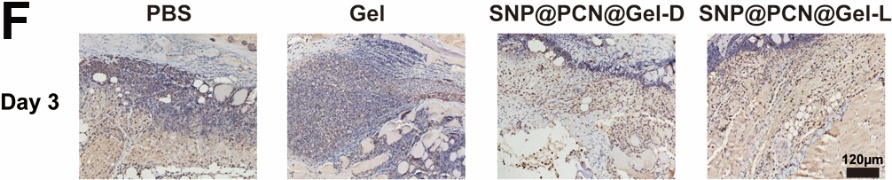


**Fig. S11.** (A) CD31, (B) VEGF, (C) α-SMA, (D) HIF-1α, (E) IL-1β and (F) IL-6 staining of skin tissue sections on day 3 post injury.
